# Potential marker genes for psoriasis revealed based on single-cell sequencing and Mendelian randomization analysis

**DOI:** 10.3389/fgene.2025.1634874

**Published:** 2025-11-17

**Authors:** Ying Dong, Hong-Song Ge, Rui-Xue Chang, Jing Chu

**Affiliations:** 1 Department of Dermatology, Anhui Provincial Children’s Hospital, Hefei, Anhui, China; 2 Department of Dermatology, The First Affiliated Hospital of University of Science and Technology of China, Hefei, Anhui, China; 3 Department of Pathology, Anhui Provincial Children’s Hospital, Hefei, Anhui, China

**Keywords:** psoriasis, single-cell RNA sequencing, Mendelian randomization, CD4 T cells, genes

## Abstract

**Background:**

Psoriasis is a chronic immune-mediated skin disorder characterized by excessive keratinocyte proliferation and localized inflammation. A comprehensive understanding of its molecular mechanisms is crucial for improving disease management and developing targeted therapies.

**Objective:**

This study aimed to investigate the molecular mechanisms underlying psoriasis by integrating single-cell RNA sequencing with Mendelian randomization (MR) analysis.

**Methods:**

Single-cell transcriptomic data from 174 skin samples (92 from psoriasis patients and 82 from healthy controls) were obtained from the GEO database. Data processing was conducted using the Seurat package, including quality control, normalization, dimensionality reduction, and cell-type annotation, ultimately identifying 11 distinct cell populations. MR analysis was then performed using summary statistics from the EBI database (n = 484,598) to assess the putative relationships between candidate genes and psoriasis risk.

**Results:**

Seven genetically informed candidate genes were identified as being significantly associated with psoriasis susceptibility. Among them, BIN2 and CAPN12 were linked to an increased risk, while genes such as CXXC5 and KLRD1 were associated with decreased risk. These genes were predominantly expressed in CD4^+^ T cells. Functional enrichment analyses, including Gene Set Enrichment Analysis (GSEA) and Gene Set Variation Analysis (GSVA), revealed their involvement in critical immune-related pathways, such as the IL-17 signaling and NOD-like receptor signaling pathways. Immune infiltration analysis demonstrated an elevated abundance of various immune cell types in psoriasis lesions. Moreover, transcription factor regulatory network analysis suggested that specific transcription factors may regulate the expression of these core genes, thereby contributing to psoriasis pathogenesis.

**Conclusion:**

By integrating single-cell RNA sequencing with MR analysis, we identified seven psoriasis-related genes (BIN2, CAPN12, CXXC5, KLRC1, KLRD1, PRF1, and SLFN5) that are highly expressed in CD4^+^ T cells. These genes hold promise as potential biomarkers for psoriasis diagnosis and as novel therapeutic targets.

## Introduction

Psoriasis is a complex, chronic inflammatory skin disease characterized by keratinocyte hyperproliferation and aberrant differentiation (Yu et al., 2020). Increasing evidence indicates that psoriasis is a systemic disorder rather than a condition limited to the skin alone (Gisondi et al., 2015). Epidemiological studies estimate a global prevalence of approximately 3%, with a gradual upward trend over recent years. While the overall incidence is similar between males and females, variations exist across subtypes—for example, psoriatic arthritis is more common in men, whereas plaque psoriasis tends to occur more frequently in women. The disease can arise at any age, with peak onset typically occurring between 20–40 and 50–60 years of age (Yan et al., 2022).

Current treatment strategies for psoriasis include topical agents, systemic therapies, and phototherapy. Topical treatments—such as corticosteroids or vitamin D analogs—are often used to reduce inflammation and scaling. In moderate-to-severe cases, systemic therapies such as oral or injectable immunosuppressants (e.g., methotrexate, biologic agents) may be employed. Phototherapy, which involves controlled exposure to ultraviolet (UV) light, also helps relieve symptoms in some patients (Ivanic et al., 2021). However, existing treatments are not curative and often fail to prevent relapse, highlighting the need for novel therapeutic approaches. The pathogenesis of psoriasis is multifactorial, involving a complex interplay between genetic predisposition, environmental triggers, and immune system dysregulation (Yu et al., 2020). A deeper understanding of the disease’s cellular landscape and molecular mechanisms is crucial for identifying novel therapeutic targets, thereby improving treatment outcomes and advancing both basic and clinical research.

Single-cell RNA sequencing (scRNA-seq) technology allows for the comprehensive analysis of gene expression, molecular features, and cellular states at the individual cell level. This approach is instrumental in identifying distinct cellular subpopulations, uncovering disease-specific biomarkers, and elucidating cellular interactions within the disease microenvironment (Zhang et al., 2024). For instance, Yao et al. (2023) applied multiple machine learning algorithms in combination with scRNA-seq and identified ADAM23 as a potential diagnostic biomarker for psoriasis. Similarly, Gao et al. (2024) integrated single-cell sequencing data with meta-analysis and revealed G3BP2 as an immune-related marker gene implicated in the development and progression of psoriasis.

While these studies provide valuable insights and highlight promising biomarker candidates, they fall short of investigating the relationships between these genes and psoriasis susceptibility. Establishing such putative causal links is essential for validating these biomarkers and understanding their mechanistic roles in disease pathogenesis.

Mendelian randomization (MR) offers a powerful approach to investigating potential putative causal relationships between biomarkers and disease phenotypes. By leveraging genetic variants as instrumental variables for specific exposures, MR enables the estimation of putative causal effects between exposure traits (e.g., gene expression levels) and disease outcomes. Because genetic variants are randomly assorted at conception, the conclusions drawn from MR analyses are inherently less prone to confounding and reverse causation, thereby enhancing the robustness of putative causal inference (Chu, 2024).

In this study, we retrieved single-cell RNA sequencing data from 174 samples—including 92 psoriasis cases and 82 healthy controls—from the GEO database. Data preprocessing and quality control were conducted using the Seurat package, followed by normalization, dimensionality reduction, and cell-type annotation, resulting in the identification of 11 distinct cell clusters in psoriasis lesions. To assess the potential putative causal associations between gene expression and psoriasis risk, we performed two-sample MR analysis using summary statistics from the EBI database, which included data from 484,598 individuals. Through the integration of scRNA-seq and MR, we identified seven genetically informed candidate genes as potential biomarkers associated with psoriasis susceptibility.

## Materials and methods

### Data acquisition

The Gene Expression Omnibus (GEO) is a publicly available gene expression database maintained by the National Center for Biotechnology Information (NCBI) in the United States. In this study, two datasets were retrieved from the GEO database to support downstream analyses. The single-cell RNA sequencing dataset GSE151177 was used for single-cell analysis. From this dataset, we selected 18 samples with complete single-cell expression profiles, including 13 psoriasis cases and 5 healthy controls. In addition, the bulk transcriptomic dataset GSE54456 was utilized for comparative gene expression analysis, comprising a total of 174 samples—92 from psoriasis patients and 82 from healthy controls. These datasets collectively provided a robust foundation for identifying cell-type-specific gene expression patterns and potential biomarkers associated with psoriasis.

The expression quantitative trait loci (eQTL) data used in this study were obtained from the eQTLGen Consortium (https://www.eqtlgen.org), specifically the Phase I whole-blood cis-eQTL meta-analysis (summary statistics). The eQTLGen Consortium is dedicated to exploring the genetic architecture of gene expression in whole blood and aims to elucidate the genetic underpinnings of complex traits (Võsa et al., 2021). While the consortium is currently in its second phase—focusing on large-scale genome-wide meta-analyses to identify blood eQTLs—our analyses rely exclusively on the Phase I resource.

We attempted to repeat the MR analyses using skin-specific cis-eQTLs from GTEx v8 (Skin—Sun Exposed [Lower leg] and Skin—Not Sun Exposed [Suprapubic]). Instruments were defined as independent cis-eQTLs after LD clumping and harmonization with the psoriasis GWAS. For most candidate genes, fewer than three independent instruments remained, precluding robust MR estimation; therefore, the skin-based MR replication could not be completed.

The outcome summary statistics used for Mendelian randomization analysis were obtained from the EBI GWAS Catalog (accession ID: GCST90038681). The European Bioinformatics Institute (EBI) database is a comprehensive repository that centralizes and standardizes genetic variation data derived from genome-wide association studies (GWAS). It encompasses extensive information on human traits and diseases, offering datasets that include single nucleotide polymorphism (SNP) identifiers, effect alleles, effect sizes, p-values, and sample sizes. The data are formatted consistently to facilitate accessibility and downstream analyses by researchers. The database is continuously updated to reflect the latest scientific discoveries and serves as a valuable resource for both basic and translational research, particularly in understanding the genetic basis of complex traits and advancing personalized medicine (Cantelli et al., 2022). In the psoriasis dataset used for this study, the summary statistics were derived from 5,427 cases and 479,171 controls, providing robust statistical power for putative causal inference.

### Single-cell data quality control

The gene expression matrix was initially imported using the Seurat package (Gribov et al., 2010). To ensure data quality, cells were filtered based on multiple parameters, including the total number of unique molecular identifiers (UMIs), the number of detected genes, and the proportion of mitochondrial gene expression. The mitochondrial gene proportion was calculated as the percentage of mitochondrial gene transcripts relative to the total gene expression per cell. A high proportion of mitochondrial gene expression is typically indicative of low RNA content and may reflect cellular apoptosis or degradation. To eliminate low-quality or dying cells, we implemented quality control based on the median absolute deviation (MAD) method. Values exceeding three MADs from the median for any given quality metric were considered outliers and excluded from further analysis. Additionally, DoubletFinder (v2.0.4) (McGi et al., 2019) was employed to identify and remove potential doublets from each sample individually. These steps collectively ensured the inclusion of only high-quality single cells for downstream analyses.

### Dimensionality reduction, clustering and annotation of single-cell data

Gene expression data were normalized using the LogNormalize method in Seurat. This approach adjusts the total expression of each cell to a common scale (10,000 transcripts per cell) by applying a scaling factor (*s*_*0*_), followed by logarithmic transformation to stabilize variance. CellCycleScoring was then used to calculate cell cycle scores, while FindVariableFeatures identified highly variable genes for downstream analysis.

To minimize gene expression variability arising from confounding factors—such as differences in mitochondrial gene content, ribosomal gene content, and cell cycle states—we employed the ScaleData function to regress out these effects. RunPCA was subsequently used to perform linear dimensionality reduction, and key principal components were selected for further analyses. To address batch effects, the Harmony algorithm was applied, enabling integration across samples. Nonlinear dimensionality reduction was then carried out using RunUMAP, which applies the Uniform Manifold Approximation and Projection (UMAP) technique for visualization in two-dimensional space.

For cell-type annotation, we referenced the CellMarker (Hu et al., 2023) and PanglaoDB (Franzén et al., 2019) databases and consulted relevant literature. This was further supplemented by automated annotation using SingleR, allowing accurate identification of cell populations and their corresponding marker genes within the analyzed tissue.

### Determine the contribution of cell subpopulations to disease

To evaluate the contribution of different cell subpopulations to disease pathogenesis, we simultaneously considered changes in cell abundance and gene expression. First, we identified characteristic genes for each cell cluster. Specifically, we conducted bulk differential gene expression analysis between the psoriasis and control groups to capture the transcriptional alterations associated with disease.

To quantify these changes, we defined a composite metric termed FCscore, which integrates both the fold change in gene expression and the proportional change in gene abundance within biological processes. For a given characteristic gene *i* in cell cluster *j*, let FCexp (i,j) represent the expression fold change, and FCprop (i,j) denote the proportional fold change in cell number. The FCscore (i,j) for gene *i* in cluster *j* is calculated as: FCscore (i,j) = √[FCexp (i,j) × FCprop (i,j)] (Jin et al., 2022).

This formulation captures the combined effect of gene expression and cellular abundance. The overall contribution of each cell subpopulation to psoriasis was then quantified as the average FCscore across all characteristic genes within the cluster.

### Mendelian randomization analysis

To identify potential instrumental variables (IVs) for Mendelian randomization (MR) analysis, we selected single nucleotide polymorphisms (SNPs) associated with each gene at a locus-wide significance threshold of P < 1 × 10^−8^. To ensure the independence of selected variants, linkage disequilibrium (LD) was assessed, and only SNPs with R^2^ < 0.001 within a 10,000 kb clumping window were retained.

We evaluated the MR-estimated effect of genetically proxied gene expression on psoriasis risk using complementary MR estimators and sensitivity tests:

1. Inverse-Variance Weighted (IVW) (random-effects): meta-analyzes SNP-specific Wald ratios to obtain an overall causal estimate.
2. MR-Egger regression: provides a pleiotropy-robust estimate under the InSIDE (Instrument Strength Independent of Direct Effect) assumption.
3. Weighted median: yields a consistent estimate if at least 50% of the total weight comes from valid instruments.
4. Weighted mode: consistent when the largest weight cluster of instruments identifies the same causal effect (ZEMPA), often exhibiting lower type I error than MR-Egger in simulations.
5. Directional horizontal pleiotropy assessment: evaluated using the MR-Egger intercept (sensitivity test rather than an estimator).

When only one SNP was available for a gene, we applied the Wald ratio. This multi-method framework enabled us to derive more robust estimates of the causal effects of cis- (and selected trans-) gene expression in whole blood on psoriasis risk. Finally, we conducted leave-one-out analyses, sequentially removing a single SNP at a time and re-estimating the MR effect to assess robustness.

### Sensitivity test analysis

We employed leave-one-out sensitivity analysis within the Mendelian randomization (MR) framework to evaluate the influence of individual genetic variants on the overall MR association between genetically proxied gene expression and psoriasis risk (Zeng et al., 2023). This procedure removes one single-nucleotide polymorphism (SNP) at a time and recalculates the pooled MR estimate from the remaining instruments. By iteratively excluding each SNP, we obtain a new point estimate with its 95% confidence interval, quantifying each variant’s contribution to the overall MR result.

This approach enables the identification of outlier SNPs that may disproportionately influence the MR estimate or indicate residual pleiotropy. Estimates from the full model (all SNPs) and from the leave-one-out models are summarized and compared; consistency across these estimates suggests that the MR findings are robust and not unduly driven by any single instrument, while discrepancies flag variants for further scrutiny.

### Gene set enrichment analysis (GSEA)

Based on the expression levels of genetically informed candidate genes, psoriasis patients were stratified into high-expression and low-expression groups. To explore the biological differences between these groups, GSEA was performed (Subramanian et al., 2005). The analysis used the Molecular Signatures Database (MSigDB, v7.0) curated canonical pathway gene sets—C2:CP:KEGG (downloaded as *c2. cp.kegg.v7.0. symbols. gmt*)—as the reference background (Liberzon, 2014). These gene sets represent curated biological pathways and molecular processes relevant to disease subtypes.

Differential pathway enrichment between the high- and low-expression groups was assessed, and gene sets with an adjusted p-value <0.05 were considered significantly enriched and ranked accordingly. GSEA is particularly useful in studies that integrate disease classification with functional biological interpretation, allowing the identification of key signaling pathways potentially associated with disease severity or molecular phenotype.

### Gene set variation analysis (GSVA)

GSVA is a non-parametric, unsupervised method used to evaluate gene set enrichment at the transcriptome level (Hänzelmann et al., 2013). Unlike traditional methods that focus on differential expression of individual genes, GSVA transforms gene-level expression data into pathway-level enrichment scores, enabling the assessment of biological process variation across samples.

In this study, gene sets were obtained from the MSigDB v7.0 for GSVA, we used the HALLMARK collection (H). The GSVA algorithm was applied to compute sample-level enrichment scores for each gene set across all samples (R GSVA package; default parameters unless otherwise noted), enabling evaluation of pathway activity differences among patient groups (e.g., high-vs. low-expression). This approach provides deeper insights into the molecular mechanisms underlying disease heterogeneity.

### Immune infiltration analysis

The CIBERSORT algorithm (Kawada et al., 2020) is a widely adopted computational method for estimating the relative proportions of immune cell types within complex tissue microenvironments. Based on the principles of support vector regression, CIBERSORT performs deconvolution analysis on bulk gene expression data to infer the composition of immune cell subtypes. The algorithm relies on a reference signature matrix containing 547 gene expression markers that distinguish 22 distinct human immune cell phenotypes, including various subsets of T cells, B cells, plasma cells, and myeloid cells.

In this study, CIBERSORT was employed to analyze the transcriptomic profiles of psoriasis patients, allowing for the quantification of immune cell infiltration across samples. Furthermore, correlation analyses were performed between the expression levels of genetically informed candidate genes and the inferred proportions of immune cell types, aiming to elucidate potential immune-related regulatory mechanisms involved in psoriasis pathogenesis.

### Transcription factor regulatory network

This study used the R package “RcisTarget” (Santana et al., 2024) to predict transcription factors. All calculations performed by RcisTarget are based on motifs. The normalized enrichment score (NES) of a motif depends on the total number of motifs in the database. In addition to the motifs annotated by the source data, we inferred further annotation files based on motif similarity and gene sequence. The first step in estimating the overexpression of each motif on a gene set is to calculate the area under the curve (AUC) for each motif-motif set pair. This was performed based on recovery curve calculations of the gene set against the ordering of the motifs. The NES of each motif is calculated based on the AUC distribution of all motifs in the gene set.

### Pseudotime analysis

Studies at the single-cell level allow one to characterize complex physiological processes and the transcriptional regulation of highly heterogeneous cell populations. These studies have led to the discovery of genes that identify specific cell subtypes, genes that mark intermediate states of biological processes, and genes that are in transition states between two different cell fates. In many single-cell studies, individual cells carry out gene expression processes in an asynchronous manner, with each cell being a moment in time of the transcriptional process being studied. Monocle introduces a strategy to sequence individual cells in pseudotime (pseudochronology), taking advantage of the asynchronous processes of individual cells to place them on trajectories corresponding to biological processes such as cell differentiation.

### Validation with RT-qPCR

Human epidermal keratinocyte (HaCaT) was obtained from Wuhan Pricella. Cultured HaCaT cells were treated with 10 ng/mL of M5 (IL-22, TNF-a, IL-17A, IL-1a, and Oncostatin M) (Pepro Tech) for 48 h (Ma et al., 2024). Untreated and treated cells were regarded as normal control (NC) groups and psoriasis cell model (M5) groups respectively. The RNA from the cell lines was extracted using TRIzol reagent (Invitrogen), and the RevertAid First-Strand cDNA Synthesis Kit (Thermo Fisher Scientific, Inc.) was used to synthesise cDNA. RT-qPCR analysis was performed using SYBR Green (Takara). The primer sequences are summarised in Table 1.

**TABLE 1:** Primer sequences.

Gene	Genbank accession	Primer sequences (5′to 3′)	Size (bp)	Annealing (°C)
Human GAPDH	2,597	GGA​GCG​AGA​TCC​CTC​CAA​AAT	197	61.6
		GGC​TGT​TGT​CAT​ACT​TCT​CAT​GG		
BIN2	51411	ACT​TCC​TTA​GTG​CAG​TCA​AAG​TG	234	60.4
		GAC​CCC​GCT​TGG​CAA​TTC​T		
CAPN12	147968	TCC​TGT​TCC​GCG​ACC​CTT​A	124	62.6
		GGC​TCA​GCA​CAG​AAC​TCA​TGG		
CXXC5	51523	CCG​AGC​GTC​GGA​ACA​AGA​G	100	62.7
		CCA​CTG​CTG​CCA​AAA​GAA​GAG		
KLRC1	3,821	AGC​TCC​ATT​TTA​GCA​ACT​GAA​CA	190	60.4
		CAA​CTA​TCG​TTA​CCA​CAG​AGG​C		
KLRD1	3,824	CAG​GAC​CCA​ACA​TAG​AAC​TCC​A	92	61.1
		GAA​ATG​AAG​TAA​CAG​TTG​CAC​C		
PRF1	5,551	GGC​TGG​ACG​TGA​CTC​CTA​AG	233	61.7
		CTG​GGT​GGA​GGC​GTT​GAA​G		
SLFN5	162394	GAG​TGT​GTT​GTA​GAT​GCA​GGA​A	104	60
		ACT​GCT​CGC​AGG​ATG​ATT​TCA		

### Statistical analysis

All statistical analyzes were performed using R language (version 4.3.0), and p < 0.05 was considered statistically significant.

## Results

**FIGURE 1:**
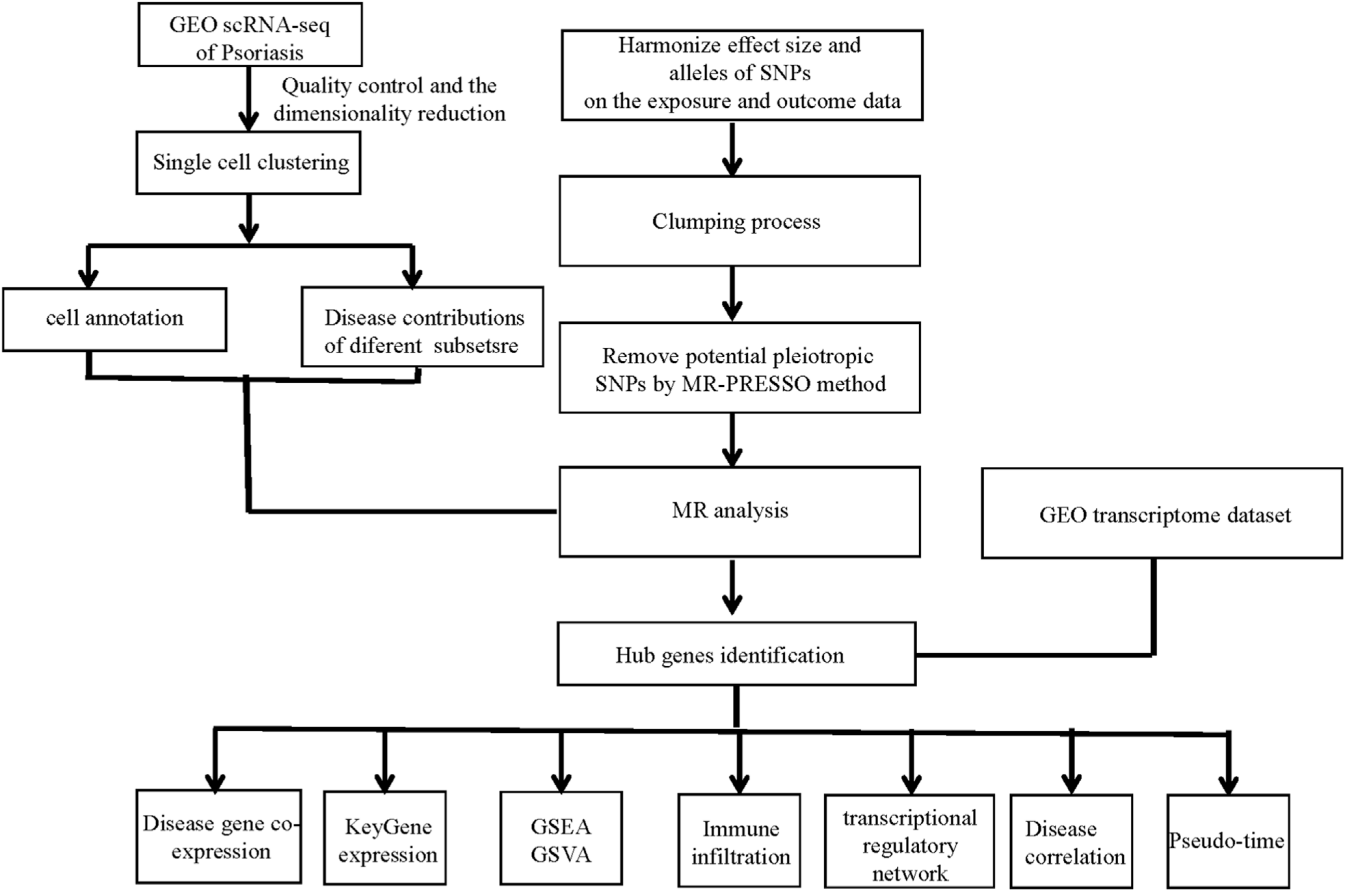
Study workflow. GEO, the Gene Expression Omnibus; scRNA-seq, Single-cell RNA sequencing; SNPs, single-nucleotide polymorphisms; MR, Mendelian randomization; MR-PRESSO, Mendelian Randomization Pleiotropy RESidual Sum and Outlier; GSEA, gene set enrichment analysis; GSVA, gene set variation analysis.

The overall design of the study was depicted in Figure 1.

### Quality control, data standardization, and cell annotation

To ensure data quality across multiple samples, cells with fewer than 200 detected genes were excluded from downstream analysis. The filtering criteria were defined as follows:

(nFeature_RNA >200 and percent. mt ≤ median +3 × MAD and nFeature_RNA ≤ median +3 × MAD and nCount_RNA ≤ median +3 × MAD and percent. ribo ≤ median +3 × MAD), where nFeature_RNA represents the number of detected genes, nCount_RNA denotes the total number of unique molecular identifiers (UMIs), percent. mt indicates the proportion of mitochondrial transcripts, and percent. ribo represents the proportion of ribosomal transcripts. In our dataset, these rules yielded the following concrete thresholds: 3,849 => nFeature_RNA≥200 and percent. mt≤14.428 and nCount_RNA≤12829.

Subsequently, the DoubletFinder (default parameters), was employed to remove potential doublets, resulting in a final dataset containing 19,670 high-quality single cells. Violin plots and scatter plots were generated to visualize the distribution of quality control metrics. Next, 2,000 highly variable genes (HVGs) were identified, from which the top 10 genes with the highest standard deviation were selected for visualization. The data were then subjected to normalization, principal component analysis (PCA), and batch effect correction using Harmony (Supplementary Figure S1).

**FIGURE 2:**
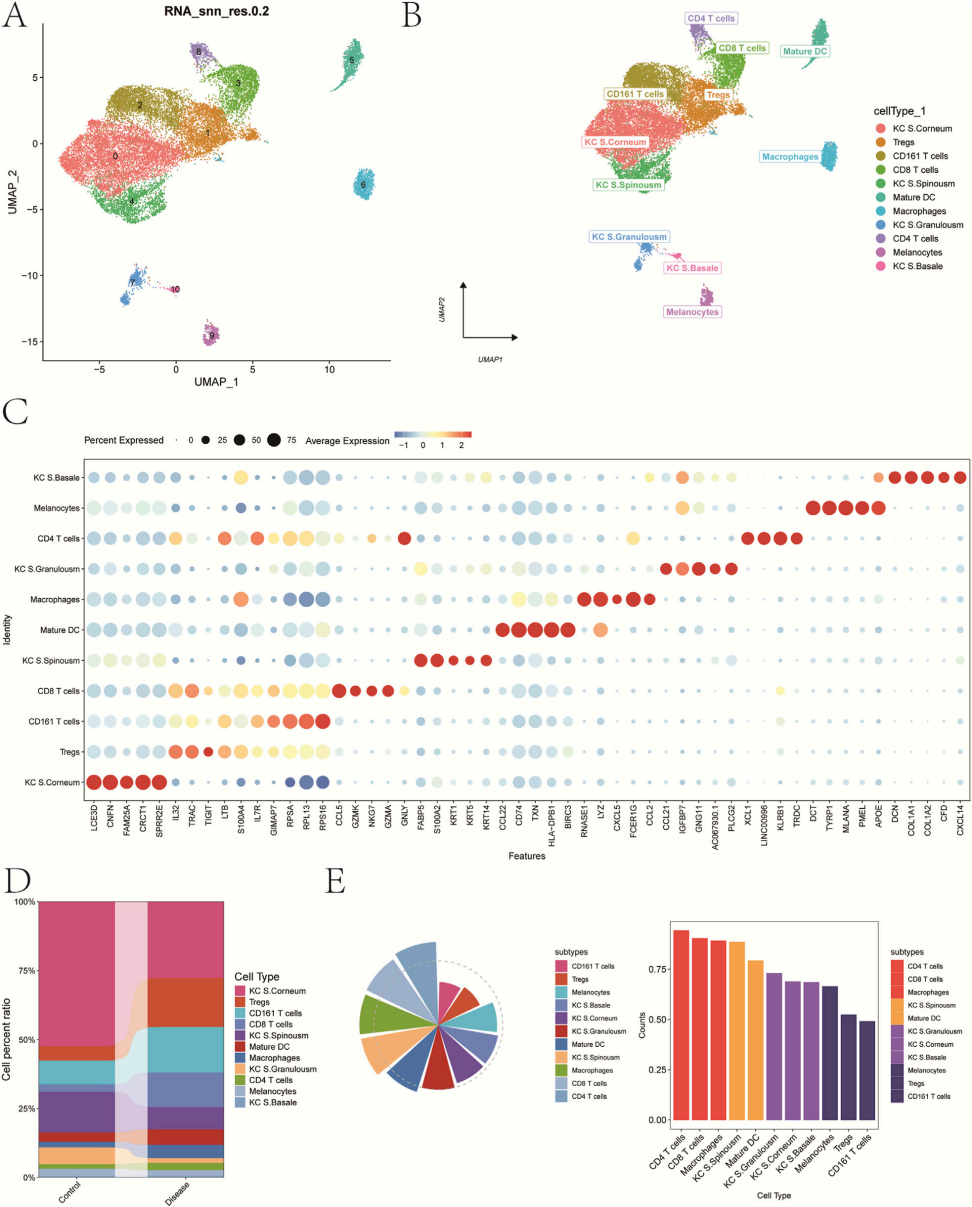
Annotation of cellular subtypes of single-cell samples. **(A)** Distribution of samples within the cell clusters. **(B)** SingleR automatically annotating the 11 cell clusters as 11 cell types. **(C)** Bubble chart of classic markers of 11 cells. **(D)** Histogram of cell proportions corresponding to groups. **(E)** Determine the contribution of cell subpopulations to psoriasis.

A total of 11 distinct cell clusters were identified following UMAP dimensionality reduction and clustering analysis (Figure 2A). These clusters were subsequently annotated based on canonical marker genes and were classified into the following cell types: keratinocytes from the stratum corneum (KC S. Corneum), regulatory T cells (Tregs), CD161^+^ T cells, CD8^+^ T cells, keratinocytes from the stratum spinosum (KC S. Spinousm), mature dendritic cells (Mature DCs), macrophages, keratinocytes from the stratum granulosum (KC S. Granulosm), CD4^+^ T cells, melanocytes, and keratinocytes from the basal layer (KC S. Basale) (Figure 2B). A bubble plot depicting the expression of classical marker genes across these 11 cell types is shown in Figure 2C, while Figure 2D presents a bar plot of cell-type proportions in psoriasis and control groups.

To investigate the contribution of each cell type to disease pathogenesis, we analyzed both cell abundance and gene expression changes. First, differentially expressed genes (DEGs) between the psoriasis and control groups were identified to reflect transcriptional alterations. Next, we introduced a composite index termed FCscore, which integrates changes in cell number and the expression levels of characteristic genes involved in disease-related biological processes. Based on this scoring system, CD4^+^ T cells were found to have the most prominent contribution to psoriasis (Figure 2E). Therefore, CD4^+^ T cells were selected as the key cell population for downstream analyses. Marker genes with log_2_ fold change >0.585 and adjusted p-value <0.05 were designated as candidate genes for subsequent investigations.

### Mendelian randomization analysis

To prioritize disease-associated candidates within the marker-gene set, we leveraged summary-level data from a large psoriasis GWAS (N = 484,598; 5,427 cases and 479,171 controls; outcome ID GCST90038681). Using the *TwoSampleMR* functions extract_instruments and extract_outcome_data, we derived genetic instruments for gene expression and the corresponding outcome associations, followed by harmonization. We then performed MR analyses to obtain MR estimates of the association between genetically proxied gene expression and psoriasis risk, which are interpreted as consistent with a causal relationship only under the standard MR assumptions (relevance, independence, and exclusion restriction).

As a result, seven significant gene-disease associations were identified, each exhibiting positive eQTL relationships, with IVW p-values <0.05.

**FIGURE 3:**
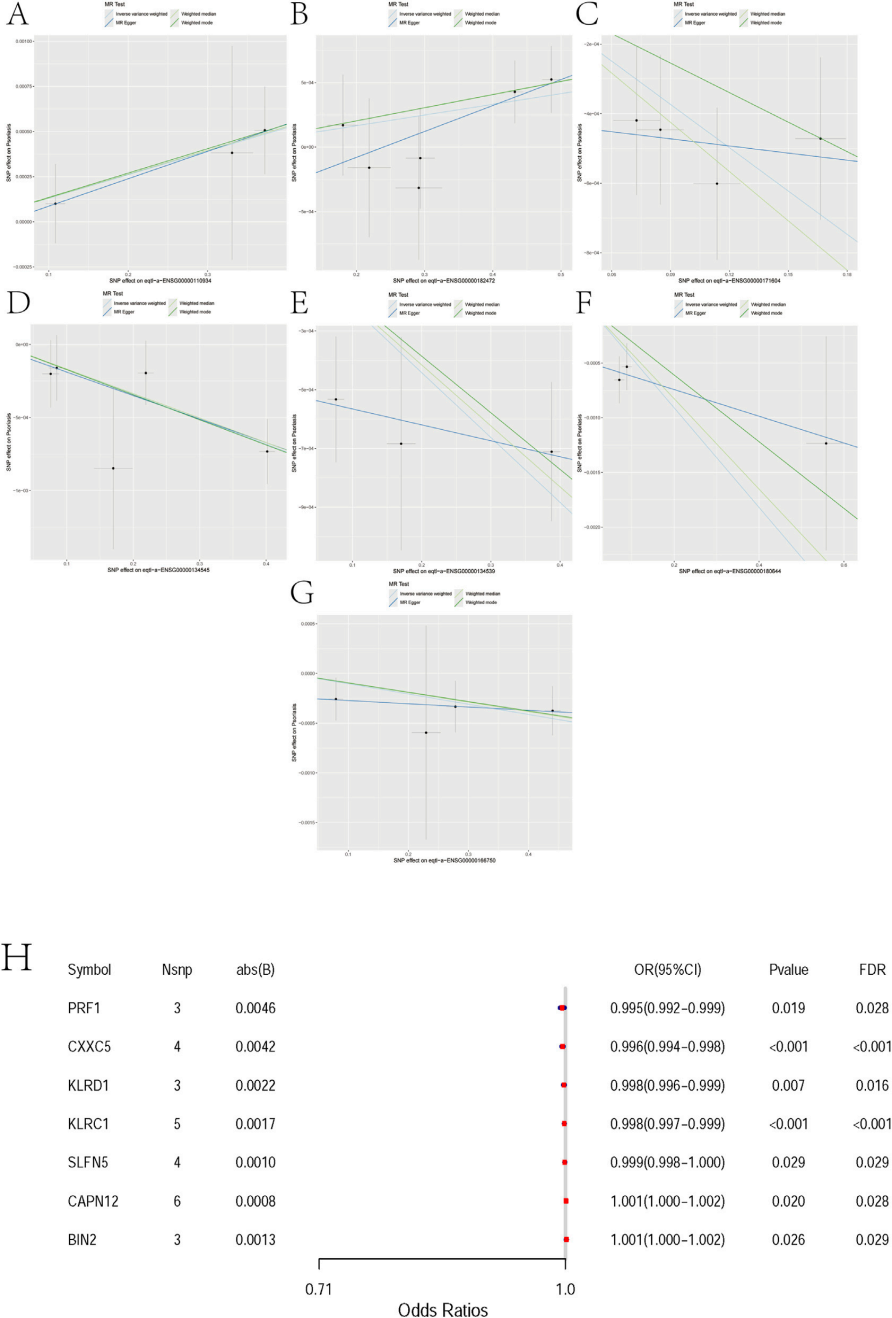
Mendelian randomization analyses between 7 pairs of Marker genes and psoriasis. **(A)** Exposure: BIN2; Outcome: Psoriasis. **(B)** Exposure: CAPN12; Outcome: Psoriasis. **(C)** Exposure: CXXC5; Outcome: Psoriasis. **(D)** Exposure: KLRC1; Outcome: Psoriasis. **(E)** Exposure: KLRD1; Outcome: Psoriasis. **(F)** Exposure: PRF1; Outcome: Psoriasis. **(G)** Exposure: SLFN5; Outcome: Psoriasis. **(H)** Mendelian randomization forest plot for candidate genes. The x-axis shows odds ratios (ORs) with 95% confidence intervals. Each row corresponds to one gene with the number of instruments (Nsnp), absolute effect size (abs(B)), nominal P value, and FDR. PRF1, CXXC5, KLRD1, KLRC1, and SLFN5 are associated with reduced risk (OR<1), whereas CAPN12 and BIN2 are associated with increased risk (OR>1). Although ORs are close to 1, most associations remain significant after FDR correction. Abbreviations: OR, odds ratio; CI, confidence interval.

For BIN2 (*Bridging Integrator 2*), three independent instruments were available (nsnp = 3). The MR estimates were directionally consistent across methods, with significant associations under IVW (OR = 1.0013; 95% CI 1.0002–1.0025; *p* = 0.026) and weighted median (OR = 1.0013; 95% CI 1.0001–1.0026; *p* = 0.037), while MR-Egger and weighted mode were not significant (Figure 3A, Supplementary File 1).

For CAPN12 (*Calpain 12*), six independent instruments were available (nsnp = 6). MR estimates were directionally consistent across methods, with nominally significant associations under IVW (OR ≈ 1.001; 95% CI ∼1.000–1.002; *p* ≈ 0.021) and weighted median (OR ≈ 1.0009; 95% CI ∼1.0002–1.0017; *p* ≈ 0.011), whereas MR-Egger and weighted mode were not significant (Figure 3B, Supplementary File 2).

For CXXC5 (*CXXC-Type Zinc Finger Protein 5*), four independent instruments were available (nsnp = 4). MR estimates were directionally consistent across methods (protective; OR < 1), with significant associations under IVW (OR = 0.9959; 95% CI 0.9940–0.9978; *p* = 2.1 × 10^−5^) and weighted median (OR = 0.9953; 95% CI 0.9929–0.9977; *p* = 1.2 × 10^−4^), whereas MR-Egger and weighted mode were not significant (Figure 3C, Supplementary File 3).

For KLRC1 (*Killer Cell Lectin Like Receptor C1*), five independent instruments were available (nsnp = 5). MR estimates were directionally consistent across methods (protective; OR < 1), with nominally significant associations under IVW (OR = 0.9983; 95% CI 0.9972–0.9994; *p* = 2.4 × 10^−4^), weighted median (OR = 0.9983; 95% CI 0.9974–0.9993; *p* = 6.9 × 10^−4^), and weighted mode (OR = 0.9983; 95% CI 0.9972–0.9994; *p* = 0.036), whereas MR-Egger was not significant (Figure 3D, Supplementary File 4).

For KLRD1(*Killer Cell Lectin Like Receptor D1*, also known as *CD94*), three independent instruments were available (nsnp = 3). MR estimates were directionally consistent across methods (protective; OR < 1), with significant associations under IVW (OR = 0.9978; 95% CI 0.9962–0.9994; *p* = 0.0067) and weighted median (OR = 0.9979; 95% CI 0.9968–0.9991; *p* = 3.7 × 10^−4^), whereas MR-Egger and weighted mode were not significant (Figure 3E, Supplementary File 5).

For PRF1 (*Perforin 1*), three independent instruments were available (nsnp = 3). MR estimates were directionally consistent across methods (protective; OR < 1), with nominally significant associations under IVW (OR = 0.9955; 95% CI 0.9917–0.9993; *p* = 0.019) and weighted median (OR = 0.9959; 95% CI 0.9930–0.9987; *p* = 0.0046), whereas MR-Egger and weighted mode were not significant (Figure 3F, Supplementary File 6).

For SLFN5 (*Schlafen Family Member 5*), four independent instruments were available (nsnp = 4). MR estimates were directionally consistent across methods (protective; OR < 1), with nominal significance under IVW (OR = 0.9990; 95% CI 0.99805–0.99989; *p* = 0.0288) and weighted median (OR = 0.9990; 95% CI 0.99812–0.99997; *p* = 0.0421), whereas MR-Egger and weighted mode were not significant (Figure 3G, Supplementary File 7).

In two-sample Mendelian randomization, we observed directionally consistent associations between several genes and disease risk that remained significant after multiple-testing correction. Specifically, PRF1 (OR = 0.995, FDR = 0.028), CXXC5 (0.996, FDR<0.001), KLRD1 (0.998, FDR = 0.016), KLRC1 (0.998, FDR<0.001), and SLFN5 (0.999, FDR = 0.029) were associated with reduced risk, whereas CAPN12 (OR = 1.001, FDR = 0.028) and BIN2 (1.001, FDR = 0.029) were associated with increased risk. Although the estimated effects were small (|β| ≤ 0.0046; Nsnp = 3–6 per gene), the associations persisted after FDR adjustment, supporting these genes as putative causal candidates. These findings indicate modest yet directionally robust genetically proxied effects that warrant further functional and clinical validation (Figure 3H).

We evaluated directional pleiotropy using the MR-Egger intercept. For all seven genes (BIN2, CAPN12, CXXC5, KLRC1, KLRD1, PRF1, SLFN5), the intercepts were near zero with non-significant *p* values (all ≥0.28), indicating no evidence of directional pleiotropy. We note that the number of instruments per gene is limited, so power for Egger is modest (Supplementary File 8).

Across the seven candidates, BIN2 and CAPN12 show risk-increasing IVW estimates (OR>1), whereas CXXC5, KLRC1, KLRD1, PRF1, and SLFN5 are protective (OR<1). Directions are broadly concordant across methods (see ‘Direction consistent across methods’), yet effect sizes are very small (ORs near unity). IVW and weighted median often reach nominal significance, while MR-Egger/weighted mode do not, consistent with limited power (nsnp = 3–6 for most genes). Together with non-significant Egger intercepts, we find no clear evidence of directional pleiotropy or substantial heterogeneity; nevertheless, results should be interpreted cautiously. Overall, these signals are best viewed as etiologic prioritization for immune-regulatory pathways rather than clinically meaningful risk effects.

To assess the robustness of these associations, leave-one-out sensitivity analysis was conducted for each gene–trait pair. The results demonstrated that exclusion of any single SNP had minimal impact on the overall effect estimates, suggesting the associations are stable and not driven by individual variants (Supplementary Figure S2). Furthermore, heterogeneity analysis showed no significant heterogeneity across the seven gene–psoriasis associations, indicating statistical consistency and supporting the validity of the MR findings (Table 2).

**TABLE 2:** Heterogeneity tests for Mendelian randomization results.

Exposure	id.exposure	Outcome	id.outcome	Method	Q	Q_df	Q_pval
BIN2	ENSG00000110934	Psoriasis	GCST90038681	MR Egger	0.00962502771199984	1	0.921847150021169
BIN2	ENSG00000110934	Psoriasis	GCST90038681	Inverse variance weighted	0.0499845624830235	2	0.975317440239049
CAPN12	ENSG00000182472	Psoriasis	GCST90038681	MR Egger	1.45185515520003	4	0.835134118981631
CAPN12	ENSG00000182472	Psoriasis	GCST90038681	Inverse variance weighted	2.41556594388285	5	0.789153811819766
CXXC5	ENSG00000171604	Psoriasis	GCST90038681	MR Egger	0.365402757172705	2	0.83301687527564
CXXC5	ENSG00000171604	Psoriasis	GCST90038681	Inverse variance weighted	1.72357221199169	3	0.631705932131266
KLRC1	ENSG00000134545	Psoriasis	GCST90038681	MR Egger	1.81068914188342	3	0.612611625829951
KLRC1	ENSG00000134545	Psoriasis	GCST90038681	Inverse variance weighted	1.82944566328652	4	0.767090912294327
KLRD1	ENSG00000134539	Psoriasis	GCST90038681	MR Egger	0.0590040165967132	1	0.808077114332275
KLRD1	ENSG00000134539	Psoriasis	GCST90038681	Inverse variance weighted	3.99460223583679	2	0.135701030539963
PRF1	ENSG00000180644	Psoriasis	GCST90038681	MR Egger	0.221456529538924	1	0.637932350008808
PRF1	ENSG00000180644	Psoriasis	GCST90038681	Inverse variance weighted	4.5642674102866	2	0.102066195046612
SLFN5	ENSG00000166750	Psoriasis	GCST90038681	MR Egger	0.0706814369647002	2	0.965276472687349
SLFN5	ENSG00000166750	Psoriasis	GCST90038681	Inverse variance weighted	0.907639472224874	3	0.823583848060954

We tested reverse causation by treating psoriasis liability as the exposure and gene expression as the outcome, using four LD-clumped instruments per gene (nsnp = 4). Effects are reported on the expression scale (β ± 95% CI; two-sided p). Across the seven genes, no robust reverse effect was detected after multiple testing except CXXC5, which remained significant (β = 15.043; p = 2.86 × 10^−4^; FDR = 0.002; Bonferroni = 0.002). Cochran’s Q indicated no substantial heterogeneity. Leave-one-out flagged SLFN5 as potentially single-instrument–driven, whereas other genes passed. Overall, these results argue against a predominant psoriasis → expression direction and are instead consistent with expression → psoriasis; CXXC5 may reflect disease-driven transcriptional feedback and warrants further validation (Supplementary File 9).

To explore the cellular distribution of these genetically informed candidate genes, we used the FeaturePlot and DotPlot functions in the Seurat R package. As shown in Supplementary Figure S3, all seven genes exhibited relatively high expression levels in CD4^+^ T cells, underscoring their potential role in the immune-mediated pathogenesis of psoriasis.

### GSEA analysis

**FIGURE 4:**
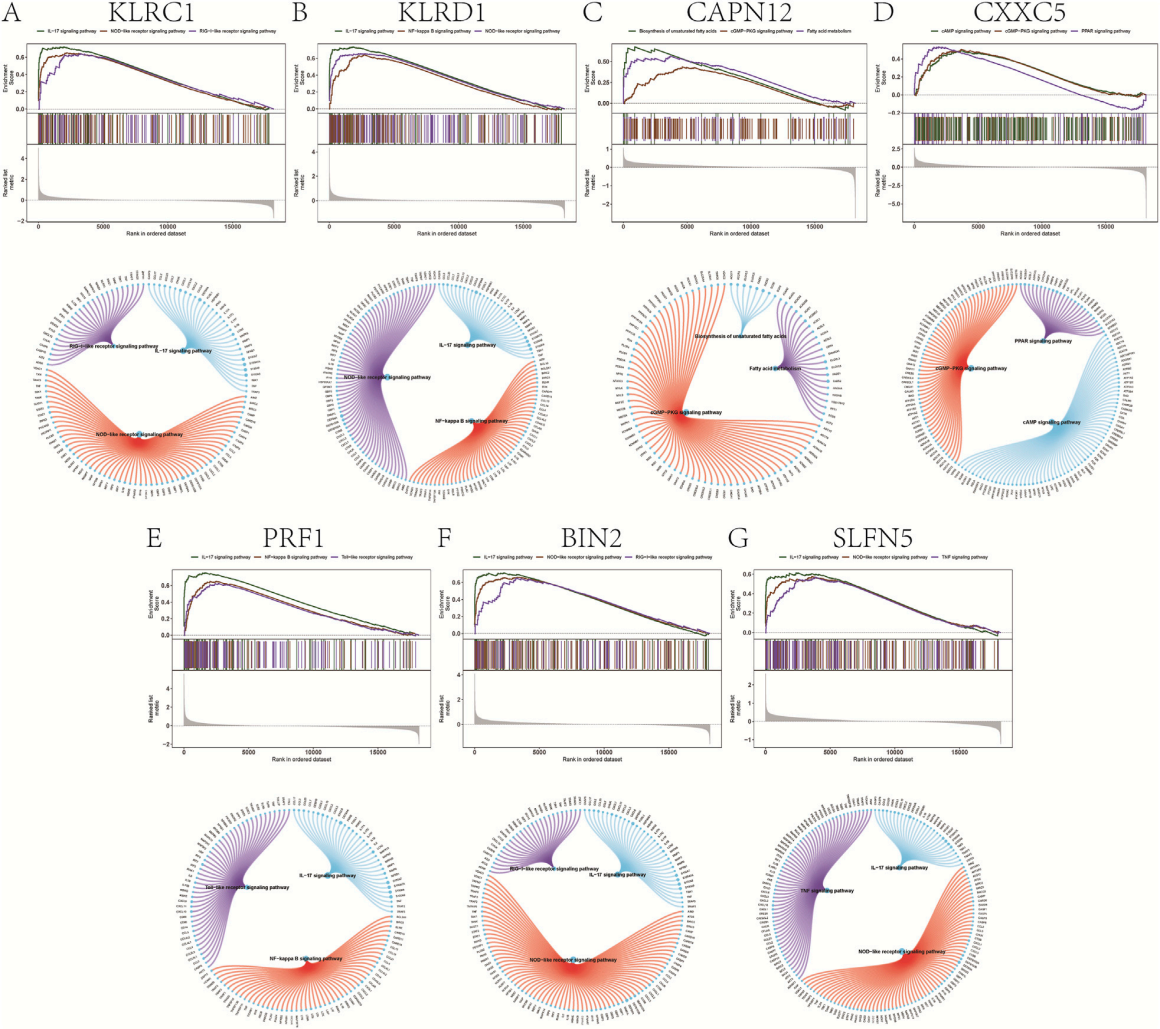
Gene set enrichment analysis. **(A–G)** Signaling pathways involved in different genetically informed candidate genes.

GSEA prioritized significantly enriched pathways by normalized enrichment score (NES) and, for each gene, displays the top three terms (Figures 4A–G). KLRC1, KLRD1, PRF1, BIN2, and SLFN5 showed enrichment concentrated in immune-inflammatory modules—including IL-17 signaling, the TNF/NF-κB axis, and innate immune sensor pathways (NOD-, RIG-I-, and Toll-like receptors)—consistent with psoriasis-relevant Th17/innate activation. By contrast, CAPN12 was primarily enriched for fatty acid metabolism/unsaturated fatty acid biosynthesis and cGMP–PKG signaling, pointing to potential immunometabolic links (e.g., epidermal lipid homeostasis, vascular tone, leukocyte trafficking). CXXC5 was enriched for cAMP and PPAR signaling, pathways implicated in keratinocyte differentiation, lipid balance, and anti-inflammatory responses, aligning with its putatively protective direction of effect. Leading-edge analyses indicated that in each panel, a compact subset of core genes drives the observed enrichment, supporting mechanistic coherence across the highlighted pathways.

### GSVA analysis

To further elucidate the functional relevance of the identified genetically informed candidate genes, GSVA was performed to assess pathway-level activity across samples. The results revealed distinct immune- and signaling-related pathway enrichments for each gene, suggesting their potential roles in modulating psoriasis progression.

**FIGURE 5:**
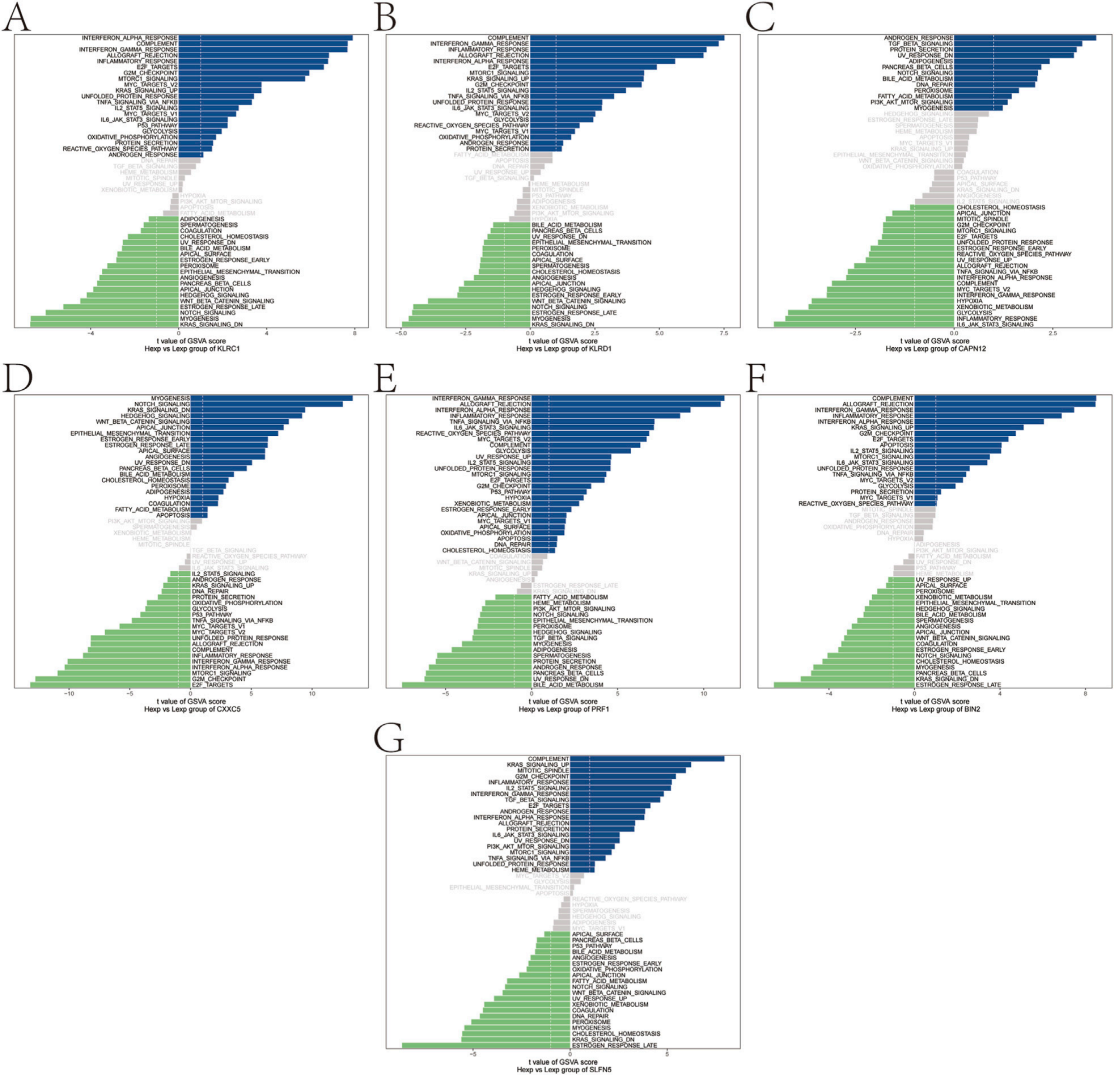
Gene set variation analysis (GSVA). **(A–G)** GSVA pathway with significant enrichment of genetically informed candidate genes: top indicates high expression and bottom indicates low expression based on hallmarker background set.

Specifically, KLRC1 was enriched in pathways such as INTERFERON_ALPHA_RESPONSE and COMPLEMENT (Figure 5A), while KLRD1 was associated with COMPLEMENT and INTERFERON_GAMMA_RESPONSE (Figure 5B).

CAPN12 was enriched in ANDROGEN_RESPONSE and TGF_BETA_SIGNALING pathways (Figure 5C), and CXXC5 showed enrichment in MYOGENESIS, NOTCH_SIGNALING, and related pathways (Figure 5D). PRF1 was enriched in INTERFERON_GAMMA_RESPONSE and ALLOGRAFT_REJECTION (Figure 5E), while BIN2 also showed enrichment in COMPLEMENT and ALLOGRAFT_REJECTION pathways (Figure 5F). Finally, SLFN5 was enriched in COMPLEMENT and KRAS_SIGNALING_UP (Figure 5G). These findings further support the hypothesis that these genetically informed candidate genes may influence psoriasis pathogenesis by modulating a range of immune responses, interferon signaling, and inflammatory signaling cascades.

### Immune infiltration

**FIGURE 6:**
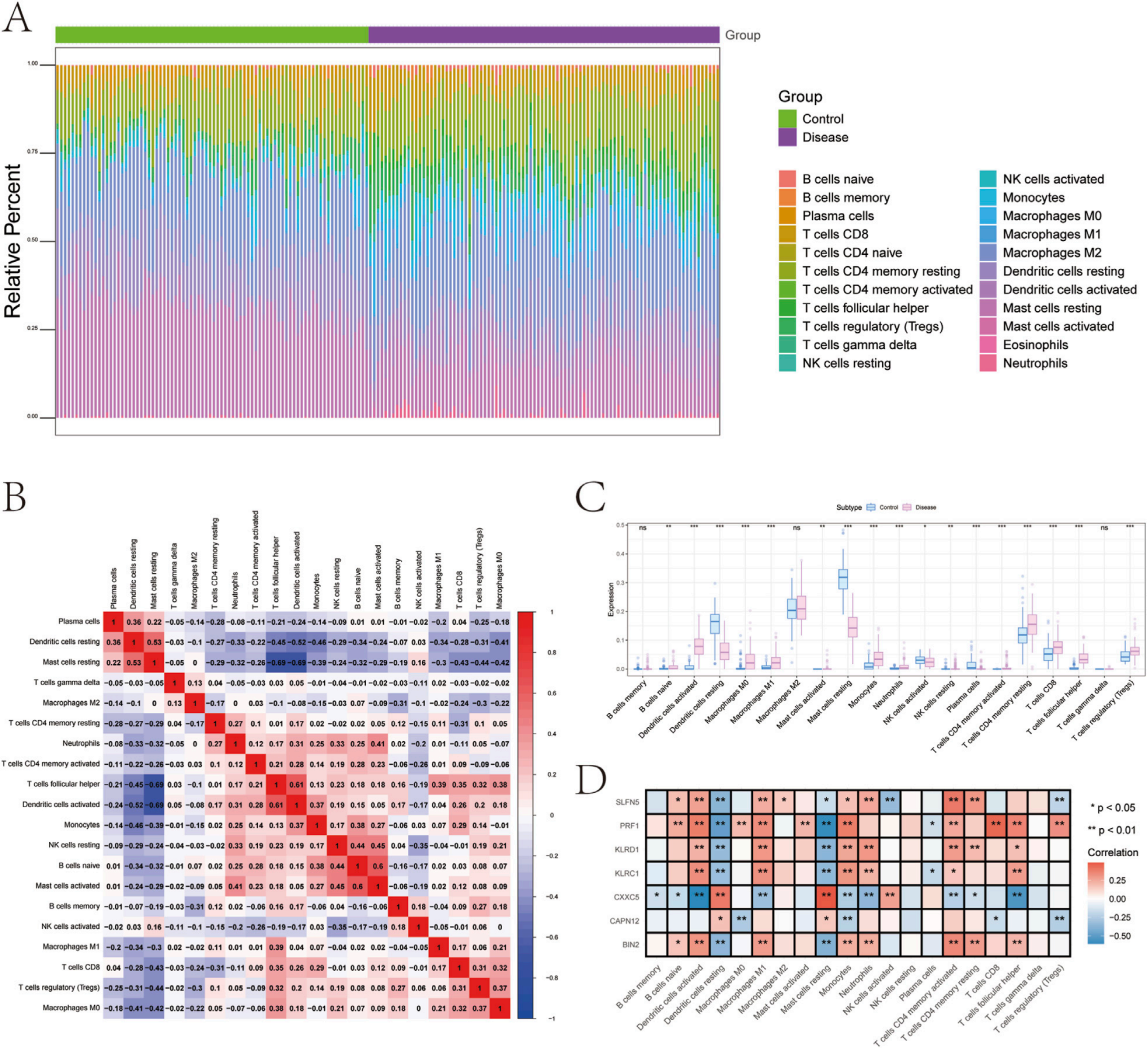
Analysis of immune cell infiltration. **(A)** Relative percentages of immune cell subpopulations. **(B)** Correlation between immune cell subpopulations. **(C)** Differences in immune cell content between psoriasis and control samples. **(D)** Correlation between 7 genetically informed candidate genes and immune cells.

The tissue microenvironment—comprising fibroblasts, immune cells, extracellular matrix components, growth factors, and inflammatory mediators—plays a pivotal role in disease pathogenesis, influencing diagnosis, prognosis, and treatment responsiveness. To investigate immune dynamics in psoriasis, we compared the immune cell infiltration landscape between disease and control groups. The distribution and intercellular correlations of infiltrating immune cells are shown in Figures 6A,B (Supplementary File 10). Compared to healthy controls, psoriasis patients exhibited significantly higher infiltration of activated dendritic cells, M0 and M1 macrophages, monocytes, neutrophils, resting CD4^+^ memory T cells, CD8^+^ T cells, follicular helper T cells, and regulatory T cells (Tregs). In contrast, resting dendritic cells, resting mast cells, activated NK cells, and plasma cells were significantly reduced in the disease group (Figure 6C).

We further examined the correlation between key gene expression and immune cell infiltration (Figure 6D, Supplementary File 11):

- KLRC1 expression was positively correlated with activated CD4^+^ memory T cells, follicular helper T cells, monocytes, M1 macrophages, activated dendritic cells, and neutrophils, but negatively correlated with plasma cells, resting dendritic cells, and resting mast cells.
- KLRD1 showed positive correlations with resting and activated CD4^+^ memory T cells, follicular helper T cells, monocytes, M1 macrophages, activated dendritic cells, and neutrophils, while negatively correlated with resting dendritic cells and mast cells.
- CAPN12 was positively correlated with resting dendritic cells and mast cells, and negatively associated with CD8^+^ T cells, Tregs, monocytes, and M0 macrophages.
- CXXC5 expression was positively associated with activated NK cells, resting dendritic cells, and mast cells, but negatively correlated with naive/memory B cells, CD4^+^ T cells, follicular helper T cells, monocytes, M1 macrophages, activated dendritic cells, and neutrophils.
- PRF1 demonstrated strong positive correlations with naive B cells, CD8^+^ T cells, activated CD4^+^ memory T cells, follicular helper T cells, Tregs, monocytes, M0/M1 macrophages, activated dendritic cells, and mast cells, but was negatively associated with plasma cells, resting dendritic cells, and mast cells.
- BIN2 expression positively correlated with naive B cells, resting and activated CD4^+^ memory T cells, follicular helper T cells, monocytes, M1 macrophages, activated dendritic cells, and neutrophils, and showed negative correlations with resting dendritic cells and mast cells.
- SLFN5 was positively associated with naive B cells, resting and activated CD4^+^ memory T cells, monocytes, M1 and M2 macrophages, activated dendritic cells, and neutrophils, but negatively correlated with Tregs, activated NK cells, resting dendritic cells, and mast cells.

These findings suggest that the identified genetically informed candidate genes are closely linked to distinct immune cell populations and may influence psoriasis progression by modulating the immune microenvironment.

### Relationship between genetically informed candidate genes and disease-related genes

**FIGURE 7:**
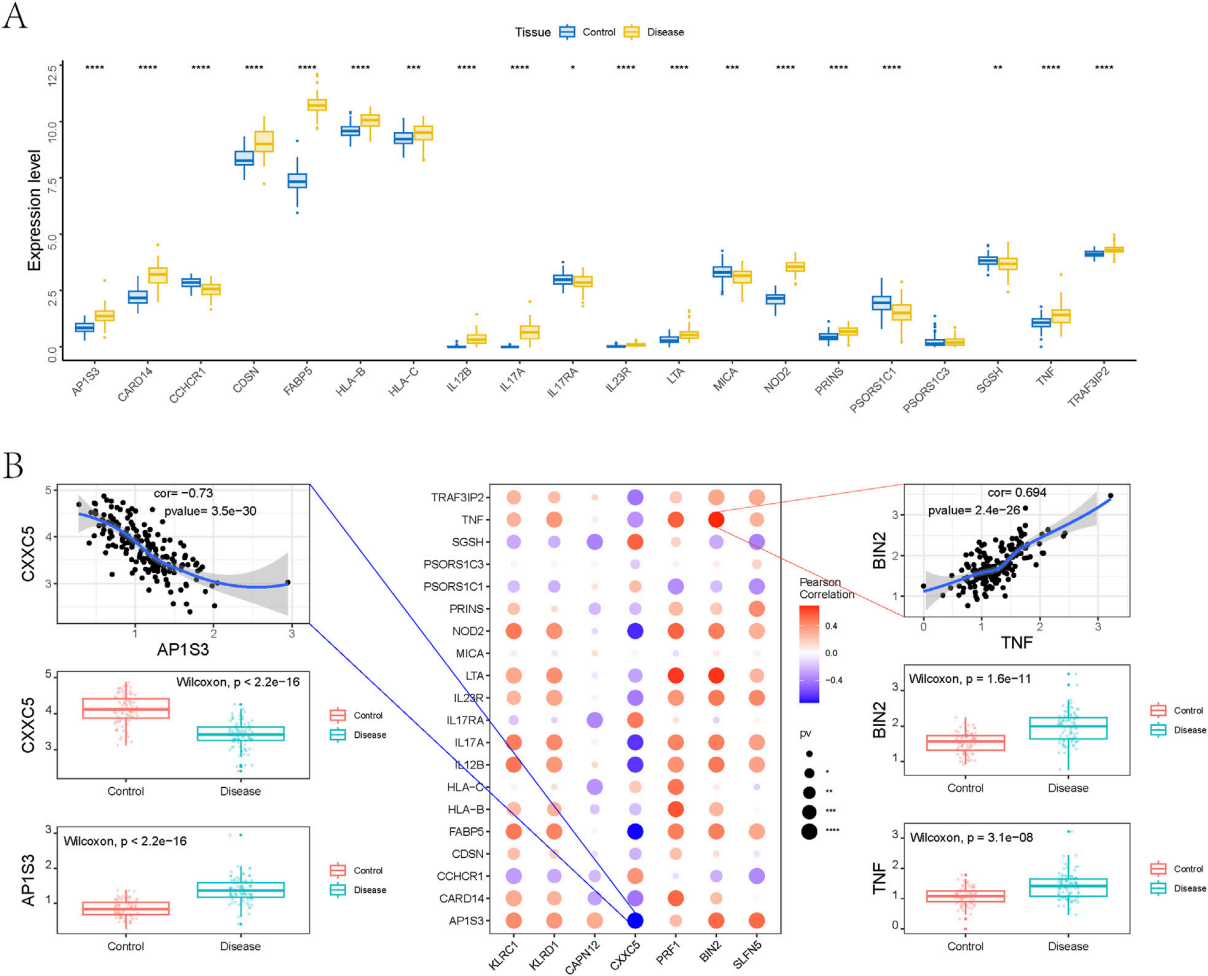
Relationship between genetically informed candidate genes and disease-related genes. **(A)** Differences in the expression of disease-related genes. **(B)** Pearson correlation analysis between genetically informed candidate genes and disease genes.

To further explore the relationship between candidate genes and known psoriasis-associated regulators, we retrieved disease-regulating genes from the GeneCards database and analyzed the expression levels of the top 20 genes ranked by Relevance*Score and transcriptomic expression. Compared to the control group, several genes—including AP1S3, CARD14, CDSN, FABP5, HLA-B, HLA-C, IL12B, IL17A, IL23R, LTA, NOD2, PRINS, TNF, and TRAF3IP2—were significantly upregulated in psoriasis samples (Figure 7A).

To examine potential interactions between our identified candidate genes and established psoriasis therapeutic targets, we performed gene co-expression analysis using bulk RNA-seq data. As shown in Figure 7B, multiple candidate genes exhibited significant correlations with immune-related targets such as TNF, IL17A, IL17RA, and IL23R. Notably, BIN2 showed a strong positive correlation with TNF (*Pearson r* = 0.694, *p* = 2.4 × 10^−26^), while CXXC5 was significantly negatively correlated with AP1S3 (*Pearson r* = −0.73, *p* = 3.5 × 10^−30^). Differential expression analysis further revealed that both BIN2 and TNF were markedly upregulated in psoriatic tissues, whereas CXXC5 and AP1S3 were downregulated, indicating potential co-expression and functional relationships in disease pathogenesis.

Next, we evaluated the expression relationships between the seven genetically informed candidate genes and the most relevant disease-regulating genes at the single-cell level. The results demonstrated that BIN2, CAPN12, CXXC5, PRF1, KLRD1, and KLRC1 were negatively correlated with IL36RN, AP1S3, PSORS1C1, TNF, and TRAF3IP2, while showing positive correlations with HLA-C. In contrast, SLFN5 was positively associated with HLA-C and TNF, but negatively correlated with the other four disease-regulating genes (Supplementary Figures S4–S10).

**FIGURE 8:**
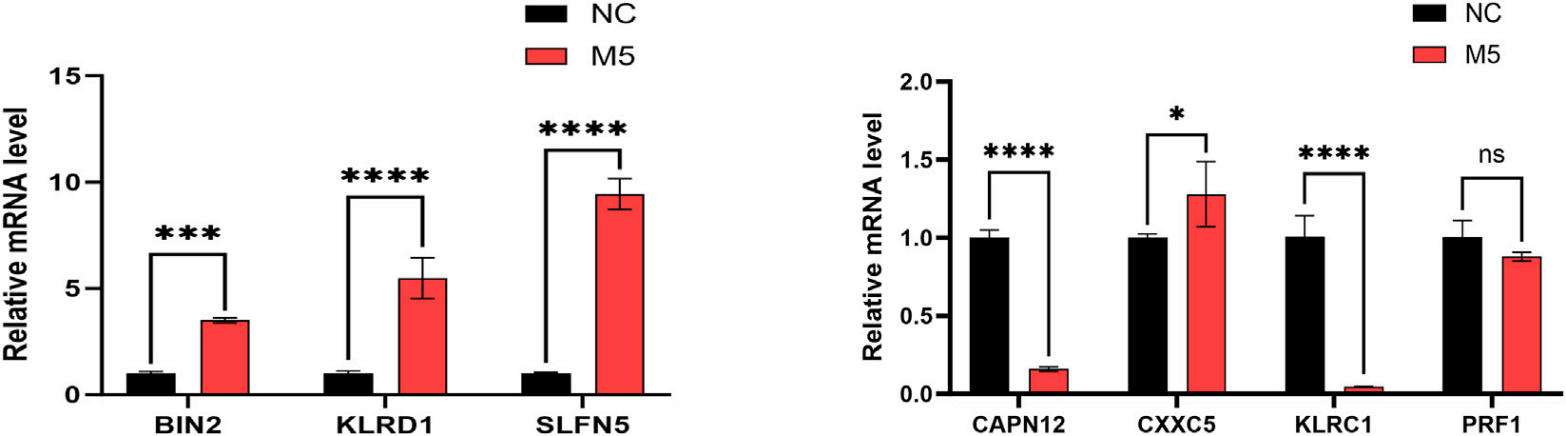
The RT-qPCR results of 7 genetically informed candidate genes. GAPDH normalization (ΔCt; 2^–ΔΔCt^), n = 3 biological replicates with technical triplicates, and one-way ANOVA with Tukey’s post hoc for group comparisons (Red represents M5 groups, black represents normal control (NC) groups. *, p < 0.05; **, p < 0.01; ***, p < 0.001).

To validate gene expression patterns, we performed qPCR to assess mRNA levels of the seven genetically informed candidate genes (BIN2, CAPN12, CXXC5, KLRC1, KLRD1, PRF1, and SLFN5) in normal control (NC) and M5-treated groups. Compared to the NC group, BIN2, KLRD1, SLFN5, and CXXC5 were significantly upregulated in the M5 group, whereas CAPN12 and KLRC1 were significantly downregulated (Figure 8). These findings suggest that the identified genetically informed candidate genes may participate in psoriasis-related inflammatory responses and potentially interact with known disease-regulating targets, providing further insight into their roles in disease progression.

### Transcription factor regulatory network

**FIGURE 9:**
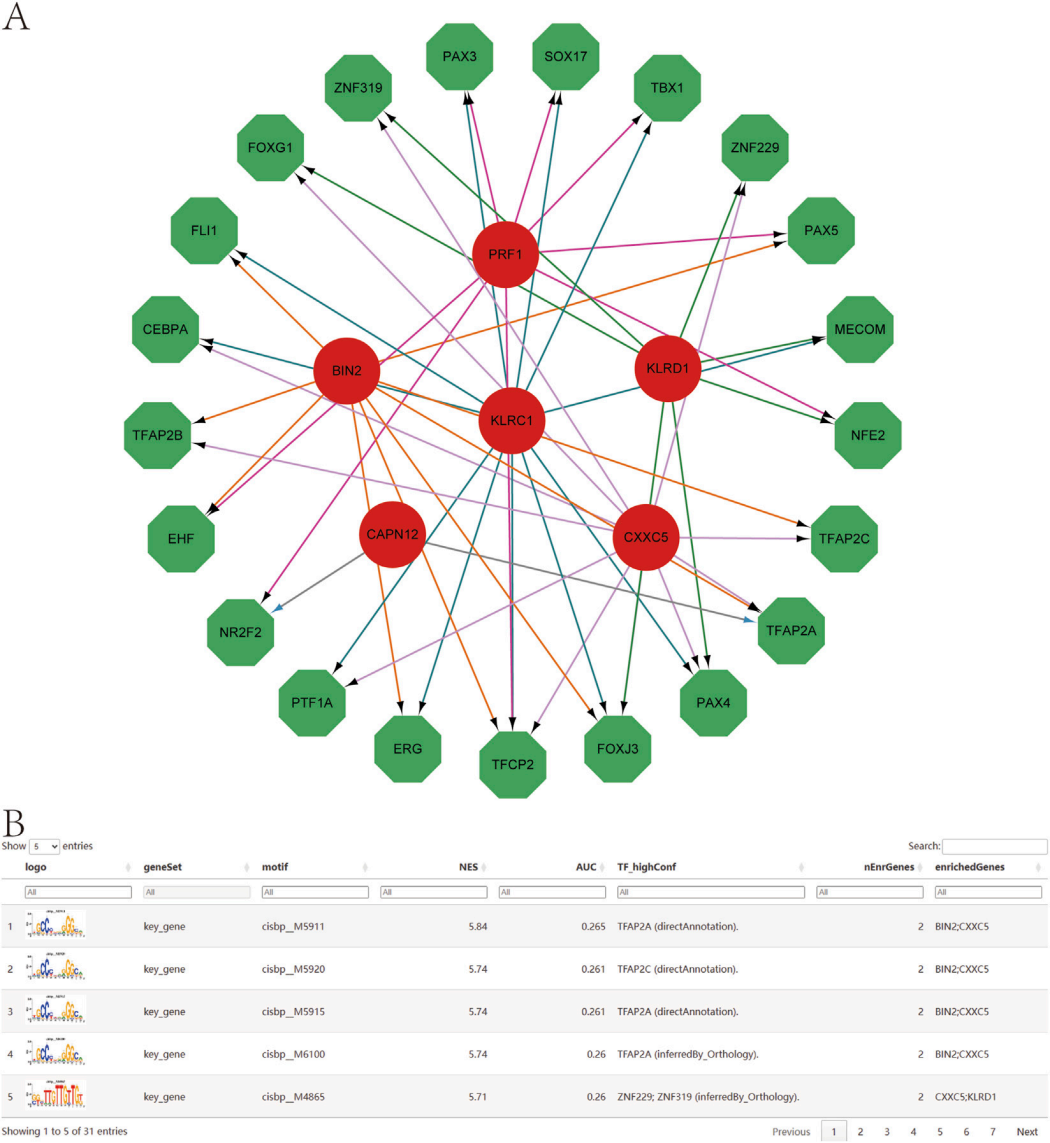
Transcription factor regulatory network. **(A)** Transcriptional regulatory networks of genetically informed candidate genes. Red indicates mRNAs and green indicates transcription factors. **(B)** Presentation of the highest AUC motif enrichment, including NES, AUC, and TF_highConf (transcription factors annotated to motifs).

Using the set of genetically informed candidate genes identified in this study, we performed transcriptional regulatory analysis and found that these genes are collectively regulated by multiple common transcription factors (TFs) (Figure 9A). To further investigate the regulatory landscape, we conducted TF enrichment analysis based on cumulative recovery curves. The results revealed that several transcription factor motifs were significantly enriched in association with the genetically informed candidate genes.

Among them, cisbp__M5911 emerged as the most significantly enriched motif, with the highest normalized enrichment score (NES = 5.84) across all tested motifs. The motif–TF annotation, combined with gene prioritization analysis, underscores its potential regulatory importance. A summary of the enriched motifs and their corresponding transcription factors associated with key gene regulation is presented in Figure 9B, providing further insights into the transcriptional control mechanisms that may influence psoriasis-related gene expression.

### Pseudotime series analysis

To investigate the dynamic changes in gene expression during cell differentiation, we first calculated the similarity between individual cells and constructed a cell differentiation trajectory using pseudotime analysis. This trajectory was visualized to depict the temporal progression of cellular states, providing insights into the development and differentiation processes. Cells were visualized and color-coded based on Pseudotime values—a probabilistic metric inferred by the Monocle algorithm that reflects the temporal ordering of cells based on their gene expression profiles—and Cell Type, which represents distinct branches or fates along the trajectory.

Because gene expression patterns often differ significantly across trajectory branches, global heatmaps may fail to capture these nuanced differences. Therefore, we identified all branch points and computed genes that exhibited marked expression differences before and after each branching event. These genes were visualized using a branch-specific heatmap, with the trajectory originating from a central “pre-branch” state and diverging into two distinct fates: “Cell fate 1” and “Cell fate 2”, representing the two major directions of differentiation. By default, differentially expressed genes were clustered into six groups based on their expression dynamics across branches.

Finally, we visualized the temporal expression patterns of the genetically informed candidate genes along the pseudotime trajectory to illustrate their potential roles during different stages of cell development (Supplementary Figure S11).

## Discussion

Psoriasis is a polygenic, immune-mediated disease closely related to genetic variations and environmental factors. It not only affects skin health but is also associated with multiple comorbidities, such as metabolic syndrome, cardiovascular diseases, and depression (Griffiths et al., 2021). In 2014, the World Health Organization (WHO) passed a resolution acknowledging psoriasis as a “chronic, non-infectious, painful, disfiguring, and disabling condition for which no cure exists.” (Griffiths et al., 2021). Currently, the absence of disease-specific, activity-specific, or outcome-specific biomarkers impedes effective disease monitoring and the development of personalized treatments. Hence, further exploration of the disease’s pathogenic mechanisms could offer novel approaches for precision medicine.

The development of single-cell RNA sequencing and machine learning in recent years has enabled the utilization of molecular genetic data to improve psoriasis diagnosis and investigate its mechanisms. This study utilized 18 samples with complete single-cell expression profiles and 174 transcriptome datasets for psoriasis from the NCBI GEO public database. After quality control, normalization, dimensionality reduction, and cell annotation, 11 distinct cellular clusters were identified, with CD4^+^ T cells showing the highest contribution to the disease. While the pathophysiology of psoriasis remains incompletely understood, the critical involvement of CD4^+^ T cells is established. Pathological activation of the TNF/IL-23/IL-17 cytokine axis drives the differentiation and activation of CD4^+^ T cells, causing their infiltration into diseased skin (Natoli et al., 2023). Consequently, this study focused on CD4^+^ T cells as primary targets for further analysis and selected relevant marker genes for candidate gene sets.

Additional Mendelian randomization analysis pinpointed seven marker genes linked to psoriasis: BIN2, CAPN12, CXXC5, KLRC1, KLRD1, PRF1, and SLFN5. BIN2 and CAPN12 are linked to an increased risk of psoriasis. BIN2 encodes a membrane-sculpting adapter protein expressed in immune cells (leukocytes), known to influence actin dynamics, podosome formation, cell motility, and phagocytosis (Sánchez-Barrena et al., 2012). Despite this role in innate immune function, BIN2 has not been prominently featured in past psoriasis research. It has appeared as an upregulated gene associated with innate immune pathways in inflammatory skin conditions (de Oliveira et al., 2022), but there are no reports of BIN2 being a psoriasis risk gene or a validated disease marker. Thus, the identification of BIN2 in the context of psoriasis likely represents a novel finding in the current study, with no prior direct association in the literature.

CAPN12 is a calcium-dependent cysteine protease expressed in the epidermis and hair follicles, implicated in skin barrier formation and keratinocyte differentiation (Bochner et al., 2016). Historically, CAPN12 has not been a well-known psoriasis gene. Apart from this study, CAPN12’s involvement was not previously reported, indicating that its association with psoriasis is a relatively novel discovery.

CXXC5, KLRC1, along with five additional genes, are linked to a reduced risk of psoriasis. CXXC5 is an epigenetic regulator that binds unmethylated DNA; it has known roles in Wnt signaling and immune regulation (Ma et al., 2017). Notably, CXXC5 is highly expressed in plasmacytoid dendritic cells (pDCs) and is crucial for robust interferon responses to TLR7/9 stimulation (pDCs are one of the initiating immune cell types in psoriasis, as self-DNA/RNA induced pDC activation has been implicated in psoriatic plaque development) (Ma et al., 2017). Despite this immune relevance, CXXC5 has not been previously linked to psoriasis in genome-wide studies or expression profiling. There are no known psoriasis GWAS hits or published functional studies for CXXC5 in this disease. Its mention in the current context is therefore likely a new finding, making CXXC5 a potentially novel gene association for psoriasis.

KLRC1 and KLRD1 encode the two subunits of the CD94/NKG2A receptor, an inhibitory receptor found on natural killer (NK) cells and certain T cells. Prior studies have indeed implicated this receptor pair in psoriasis immunopathology. For example, one study on new-onset psoriasis patients found a downregulation of CD94/NKG2A on circulating NK cells, presumably reducing inhibitory signals and thus potentially heightening NK cell activity (Son et al., 2009). Conversely, in chronic plaque psoriasis, T cells in blood and lesions were reported to express higher levels of CD94/NKG2A (and other NK receptors) than normal, suggesting an abnormal expression of NK receptors on T cells (Son et al., 2009). These findings indicate that KLRC1/KLRD1 are involved in the psoriasis immune response (likely as part of dysregulated cytotoxic cell function or feedback mechanisms). Because of such prior evidence, KLRC1 and KLRD1 are not novel to psoriasis: they have been studied and recognized as part of the disease’s immune dysregulation.

PRF1 encodes perforin, a key cytolytic protein used by CD8^+^ T cells and NK cells to kill target cells. There is substantial prior evidence linking perforin to psoriasis. Immunohistochemistry studies showed significantly elevated perforin expression in psoriatic lesions (especially within the epidermis) compared to non-lesional or healthy skin (Kastelan et al., 2004). This upregulation of perforin in lesional skin suggests active involvement of cytotoxic lymphocytes in forming psoriatic plaques. Additionally, patients with severe psoriasis have higher proportions of perforin-positive CD8^+^ T cells in blood than those with mild disease, consistent with an enhanced cytotoxic immune response in more active disease (Prpić Massari et al., 2007). Overall, PRF1 has already been implicated in psoriasis pathogenesis through these expression and functional studies, so its presence in the current gene list is not unexpected or novel, but rather corroborates known immune mechanisms in psoriasis (Sidore et al., 2020).

SLFN5 is a member of the Schlafen family, a group of interferon-regulated genes involved in cell growth and immune modulation. SLFN5 has only recently been associated with psoriasis. A 2019 proteomic study (Sobolev et al., 2022) identified SLFN5 among a set of new disease-associated proteins in psoriasis, alongside other markers (ATM, ZNF512, SPATA13, etc.). That study, which used a high-throughput SomaScan assay on patient serum, reported SLFN5 as one of the proteins elevated in psoriasis patients–particularly tied to TNF-α and interferon-driven inflammation. Aside from this report, SLFN5 has not been widely mentioned in earlier psoriasis literature. Its implication appears to be part of emerging research into systemic biomarkers. Thus, SLFN5 is an already implicated gene but in a very recent context, representing a relatively novel finding that the current study is likely expanding upon.

The seven genes span established–emerging–novel tiers: PRF1 and KLRC1/KLRD1 are established in psoriasis immunity; SLFN5 is a recently reported systemic inflammatory marker; BIN2/CXXC5/CAPN12 lack prior links, suggesting novelty. Our findings corroborate known immune axes and extend them with BIN2/CXXC5/CAPN12 as new leads.

We interpret our MR findings under the standard instrumental-variable assumptions—relevance, independence, and exclusion restriction. We used ±1 Mb cis-eQTLs as instruments, applied LD clumping and allele harmonization, and reported estimates from IVW, weighted median, MR-Egger, and weighted mode (using the Wald ratio when *nsnp* = 1). We also performed sensitivity diagnostics, including the MR-Egger intercept (directional horizontal pleiotropy), Cochran’s Q (heterogeneity), and leave-one-out analyses. In addition, to probe bidirectionality, we conducted reverse MR (psoriasis liability → gene expression).

Across the seven candidates, BIN2 and CAPN12 show risk-increasing effects in the IVW analysis, whereas CXXC5, KLRC1, KLRD1, PRF1, and SLFN5 are protective; in all cases, the effect sizes are very small (OR ≈ 1.00). Accordingly, we treat these signals as etiologic prioritization leads rather than clinically actionable effects. Reverse MR revealed no robust psoriasis→expression effect, with the sole exception of CXXC5, supporting expression→psoriasis as the predominant direction and suggesting that CXXC5 may reflect disease-driven transcriptional feedback. Taken together with the MR-Egger intercepts, Cochran’s Q, and leave-one-out results, we find no clear evidence of directional pleiotropy or substantial heterogeneity, while still interpreting the findings cautiously.

We acknowledge that statistical power is limited: most genes have only 3–6 instruments (nsnp), cis-eQTLs explain modest variance in expression, true effects are very small (OR ≈ 1.000–1.005), and the cross-tissue design (using whole-blood eQTLs for a skin disease) can bias estimates toward the null. Future work will revisit MR using skin and single-cell eQTL resources and undertake mechanistic validation in disease-relevant cell types to further test and refine these genetically prioritized leads.

The convergence of our genetic findings with established therapeutic pathways carries important clinical implications. Notably, strong expression correlations were observed between several candidate genes and key inflammatory mediators central to psoriasis pathogenesis. For instance, BIN2 expression showed a close association with TNF levels, while other candidates demonstrated co-variation with IL-17A expression. These relationships suggest that the identified genes are embedded within the core cytokine-driven inflammatory network of psoriasis.

This alignment with clinically validated therapeutic targets, such as TNF and IL-17A, not only reinforces the biological relevance of these candidate genes but also highlights their potential as biomarkers of disease activity and adjunctive therapeutic targets. Given that anti-TNF and anti-IL-17A therapies have shown high efficacy in treating psoriasis, our findings support the rationale for further investigation into the mechanistic roles of these novel genes within the cytokine-regulatory axis, as well as their utility in improving patient stratification and guiding personalized treatment strategies (Gupta et al., 2021).

The co-expression of our candidates alongside TNF/IL-17A suggests that modulating these genes could influence the same pathogenic circuits targeted by biologics, potentially enhancing or refining therapeutic outcomes. Moreover, the Mendelian randomization evidence adds a crucial translational dimension: by implicating these genes as causal contributors to psoriasis risk, it bolsters the rationale for therapeutic targeting rather than considering them mere downstream epiphenomena. Notably, drug targets supported by human genetic evidence (as is the case for our candidates) have markedly higher success rates in clinical development (Minikel et al., 2024), underscoring the promise of these findings.

Several of the identified genes (e.g., KLRC1, KLRD1, PRF1) also highlight the involvement of cytotoxic lymphocyte pathways in psoriasis–an avenue not directly addressed by current cytokine-centric therapies but evident in lesional inflammation (perforin-expressing cells are known to be enriched in psoriatic epidermis (Kastelan et al., 2004). This insight raises the possibility of novel interventions that temper cell-mediated tissue damage in concert with suppressing key cytokines. In summary, by integrating single-cell transcriptomics with genetic causality, we have pinpointed seven candidate genes whose influence spans fundamental inflammatory pathways and therapeutic targets. These results not only deepen our understanding of psoriasis pathogenesis but also provide a springboard for developing more precise biomarkers and innovative treatment strategies, potentially accelerating their translation into high-impact clinical advances.

Although fatty acid metabolism and cGMP–PKG signaling (CAPN12) may seem peripheral to psoriasis, both map onto clinically observed immunometabolic and vascular phenotypes. Lipid remodeling in keratinocytes can alter barrier lipids (e.g., ceramides and polyunsaturated fatty acids), shaping eicosanoid production and amplifying IL-17/TNF-driven inflammation, thereby contributing to barrier dysfunction and epidermal hyperproliferation (Berdyshev, 2024). In parallel, cGMP–PKG signaling is a canonical regulator of vascular smooth muscle tone and endothelial responses, offering a mechanistic route to the vasodilation/erythema typical of psoriatic lesions and potentially influencing leukocyte trafficking. Conversely, the enrichment of PPAR and cAMP pathways (CXXC5) aligns with programs that promote keratinocyte terminal differentiation and lipid homeostasis (PPARα/γ) and dampen NF-κB-mediated inflammation (cAMP/PKA), consistent with a protective genetic direction and with clinical improvements in barrier integrity and reduced hyperproliferation (Kim and Choi, 2023). Taken together, these enrichments do not undermine disease relevance; rather, they highlight immunometabolic and differentiation axes that plausibly connect our genetic findings to hallmark clinical features of psoriasis (barrier compromise, acanthosis, and vascular dilation), while motivating targeted functional validation in keratinocytes and endothelial/immune compartments.

Immune infiltration studies further reveal a strong positive correlation between the five genes (BIN2, KLRC1, KLRD1, PRF1, SLFN5) and CD4^+^ T cells. To further validate the biological relevance of the seven candidate psoriasis marker genes identified in this study, we systematically reviewed and integrated research evidence from the past 5 years concerning the expression and functional regulation of BIN2, CAPN12, CXXC5, KLRC1, KLRD1, PRF1, and SLFN5 in CD4^+^ T cells.

BIN2 is categorized as an immune cell-enriched gene, with high transcriptional expression reported in T cells and NK cells, although its direct functional role in CD4^+^ T cells remains unconfirmed (Yu et al., 2025). CAPN12, on the other hand, exhibits minimal expression in immune cells, with limited evidence linking it to tumor proliferation rather than immune regulation, implying a likely non-immune-specific role (Xia et al., 2023).

KLRC1 and KLRD1 encode the inhibitory receptor NKG2A and its dimerization partner CD94, respectively, and were originally thought to be predominantly expressed in natural killer (NK) cells. However, multiple studies have demonstrated that under pathological conditions such as chronic infection, cancer, and inflammation, activated CD4^+^ T cells can also induce the expression of NKG2A/CD94 heterodimers, particularly in Th1-skewed immune responses (Gra et al., 2007; Chang et al., 2025). These NKG2A/CD94-expressing CD4^+^ T cells often exhibit cytotoxic phenotypes or terminal differentiation characteristics and are closely associated with immune exhaustion in various viral infections and autoimmune diseases. This evidence suggests that NKG2A^+^ CD4^+^ T cells may play a key immunoregulatory role in the pathological activation of CD4^+^ T cells in psoriasis (Baird et al., 2023).

PRF1 is a classical cytotoxic effector molecule and has been widely recognized as a hallmark of cytotoxic CD4^+^ T cells (CD4^+^ CTLs) across various pathological contexts. It primarily functions by releasing perforin through the granule exocytosis pathway, mediating the lysis and clearance of MHC class II^+^ target cells. PRF1^+^ CD4^+^ T cell subsets have been detected in settings such as chronic infections, malignancies, and COVID-19. These cells play a critical role in enhancing immune clearance and antigen elimination, particularly in antiviral and antitumor immunity, where they exhibit potent cytolytic capacity. However, under conditions of immune dysregulation or chronic inflammation, excessive activation of PRF1^+^ CD4^+^ T cells may also contribute to tissue damage and immune-mediated pathology. This dual role highlights their capacity to mediate both protective immunity and pathological immune responses (Oh and Fong, 2021).

CXXC5 and SLFN5 represent two opposing epigenetic regulatory mechanisms that may exert contrasting effects on transcriptional regulation in CD4^+^ T cells. CXXC5 functions as a transcriptional activator, promoting the expression of exogenous genes such as HIV-1, and may interfere with transcriptional regulatory networks to suppress genetically informed candidate genes associated with CD4^+^ T cell helper functions, thereby modulating their immune effector state (Chen et al., 2023). In contrast, SLFN5 is highly expressed in resting CD4^+^ T cells and maintains cellular quiescence and antiviral defense by inhibiting the recruitment of RNA polymerase II and inducing H3K27me3-mediated histone modifications that silence transcription (Ding et al., 2022). This antagonistic regulatory pattern suggests that CXXC5 and SLFN5 may play complementary roles in balancing CD4^+^ T cell activation and homeostasis, highlighting their potential functional relevance in psoriasis and other immune-related diseases.

In summary, among the seven candidate genes identified in this study, there is robust literature evidence supporting the expression and immunological functions of KLRC1, KLRD1, PRF1, SLFN5, and CXXC5 in CD4^+^ T cells. These genes are primarily implicated in key immune processes, including cytotoxic activity, terminal differentiation, and epigenetic regulation. Such evidence not only strengthens the biological plausibility of our Mendelian randomization results but also indicates that these genes may serve as critical immunoregulatory mediators in CD4^+^ T cell–driven inflammatory responses associated with psoriasis. Further *in vitro* and *in vivo* experiments are warranted to elucidate their mechanistic roles and evaluate their potential as diagnostic biomarkers or therapeutic targets.

Nevertheless, a major limitation of our study lies in the absence of clinical stratification, which stems from the lack of individual-level metadata (e.g., sex, age, and psoriasis subtype) in the publicly available transcriptomic datasets (GSE151177 and GSE54456). Given the well-documented heterogeneity of psoriasis, subgroup-specific analyses would likely yield more nuanced insights into gene expression dynamics and differential therapeutic responses. Consequently, we have exercised caution in extrapolating our findings to the broader psoriasis population.

Future investigations incorporating clinical cohorts with comprehensive phenotypic annotations will be essential for validating and refining the identified gene signatures across distinct patient subgroups. Stratified validation would not only enhance the biological and clinical relevance of our findings but also improve their translational potential. Furthermore, integrating gene expression data with therapeutic response profiles (e.g., to IL-17 or TNF inhibitors) could facilitate the identification of novel predictive biomarkers and advance personalized treatment strategies in psoriasis.

## Conclusion

In this study, we applied single-cell RNA sequencing to psoriatic skin lesions and identified 11 distinct cell subpopulations, among which CD4^+^ T cells exhibited the highest disease relevance. By integrating whole blood eQTL data from the EBI database with skin lesion–derived transcriptomic profiles, we conducted Mendelian randomization analysis and uncovered seven putative gene–trait associations involving BIN2, CAPN12, CXXC5, KLRC1, KLRD1, PRF1, and SLFN5. These marker genes not only show potential as diagnostic biomarkers but also offer a theoretical framework for exploring the genetic and immunological mechanisms underlying psoriasis pathogenesis.

Our findings build upon previous biomarker discovery efforts by incorporating a genetic instrument–based inference framework, providing new insights into genetically regulated pathways associated with psoriasis. However, several limitations should be acknowledged. Notably, the integration of whole-blood eQTL data with skin-derived expression profiles introduces cross-tissue inference, which may be affected by tissue- and cell type–specific regulatory mechanisms. While skin is the most disease-relevant tissue for psoriasis, current skin eQTL resources provided insufficient independent instruments for several target genes, limiting MR feasibility. We therefore prioritized the well-powered whole-blood eQTLs and explicitly acknowledge the cross-tissue limitation; future analyses will revisit skin-based MR as larger, better-powered skin eQTL datasets become available.

Additionally, while gene expression patterns were preliminarily validated in a cell-line model, clinical validation using patient-derived lesional skin samples has not yet been performed due to limited sample access and pending ethical approval. Once obtained, we plan to conduct RT-qPCR–based validation in clinical tissues. Moreover, the precise biological functions of the identified genes remain to be elucidated through further *in vitro* and *in vivo* functional studies.

In summary, this study highlights a set of genetically informed candidate genes with putative etiologic relevance to psoriasis and underscores the value of integrating single-cell transcriptomics with genetic causal inference to advance our understanding of complex immune-mediated diseases.

## Data Availability

The original contributions presented in the study are included in the article/Supplementary Material, further inquiries can be directed to the corresponding author.

## Supplementary material

SUPPLEMENTARY FIGURE 1 Quality control and data standardization of scRNA-seq data. **(A)** Quality control for each cell in scRNA seq data, displaying the number of nCount_RNA, nFeature_RNA, percent.mt, and percent.ribo. **(B)** Scatter plots showing the correlation between filtered data nCount_RNA and nFeature_RNA, percent. mt, percent.ribo. **(C)** Variance analysis to select highly variable genes in the samples (red dots represent highly variable genes, black dots represent invariant genes). **(D)** Fragmentation maps for principal component screening. **(E)** Principal component analysis (PCA). **(F)** Harmonization analysis.

SUPPLEMENTARY FIGURE 2 Mendelian randomization leave-one-out sensitivity analysis. **(A)** BIN2 on Psoriasis. **(B)** CAPN12 on Psoriasis. **(C)** CXXC5 on Psoriasis. **(D)** KLRC1 on Psoriasis. **(E)** KLRD1 on Psoriasis. **(F)** PRF1 on Psoriasis. **(G)** SLFN5 on Psoriasis.

SUPPLEMENTARY FIGURE 3 Key genes expression abundance. The FeaturePlot **(A)** and Dotplot **(B)** functions were used to view the expression of key genes in single cells.

SUPPLEMENTARY FIGURE 4 Co-expression analysis of CXXC5 with disease-related genes in single-cell sequencing samples. Scatter plots represent pearson correlation analysis between co-expressed genes.

SUPPLEMENTARY FIGURE 5 Co-expression analysis of PRF1 with disease-related genes in single-cell sequencing samples. Scatter plots represent pearson correlation analysis between co-expressed genes.

SUPPLEMENTARY FIGURE 6 Co-expression analysis of KLRD1 with disease-related genes in single-cell sequencing samples. Scatter plots represent pearson correlation analysis between co-expressed genes.

SUPPLEMENTARY FIGURE 7 Co-expression analysis of KLRC1 with disease-related genes in single-cell sequencing samples. Scatter plots represent pearson correlation analysis between co-expressed genes.

SUPPLEMENTARY FIGURE 8 Co-expression analysis of BIN2 with disease-related genes in single-cell sequencing samples. Scatter plots represent pearson correlation analysis between co-expressed genes.

SUPPLEMENTARY FIGURE 9 Co-expression analysis of SLFN5 with disease-related genes in single-cell sequencing samples. Scatter plots represent pearson correlation analysis between co-expressed genes.

SUPPLEMENTARY FIGURE 10 Co-expression analysis of CAPN12 with disease-related genes in single-cell sequencing samples. Scatter plots represent pearson correlation analysis between co-expressed genes.

SUPPLEMENTARY FIGURE 11 Pseudo-time series analysis. **(A)** Pictures of cells colored by Pseudotime values. **(B)** Pictures of cells colored by Cell Type. **(C)** Branch heatmap. **(D)** The expression changes of key genes at different periods of cell development.

SUPPLEMENTARY FILE 1 Method-wise MR summary for BIN2. IVW, weighted median, MR-Egger, and weighted mode estimates are reported using nsnp = 3 independent instruments (β, SE, OR with 95% CI, and *p*).

SUPPLEMENTARY FILE 2 Method-wise MR summary for CAPN12. IVW, weighted median, MR-Egger, and weighted mode estimates are shown using nsnp = 6 independent instruments (β, SE, OR with 95% CI, and *p*).

SUPPLEMENTARY FILE 3 Method-wise MR summary for CXXC5. IVW, weighted median, MR-Egger, and weighted mode estimates are shown using nsnp = 4 independent instruments (β, SE, OR with 95% CI, and *p*).

SUPPLEMENTARY FILE 4 Method-wise MR summary for KLRC1. IVW, weighted median, MR-Egger, and weighted mode estimates are shown using nsnp = 5 independent instruments (β, SE, OR with 95% CI, and *p*).

SUPPLEMENTARY FILE 5 Method-wise MR summary for KLRD1. IVW, weighted median, MR-Egger, and weighted mode estimates are shown using nsnp = 3 independent instruments (β, SE, OR with 95% CI, and *p*).

SUPPLEMENTARY FILE 6 Method-wise MR summary for PRF1. IVW, weighted median, MR-Egger, and weighted mode estimates are shown using nsnp = 3 independent instruments (β, SE, OR with 95% CI, and *p*).

SUPPLEMENTARY FILE 7 Method-wise MR summary for SLFN5. IVW, weighted median, MR-Egger, and weighted mode estimates are shown using nsnp = 4 independent instruments (β, SE, OR with 95% CI, and *p*).

SUPPLEMENTARY FILE 8 Directional horizontal pleiotropy was assessed using the MR-Egger intercept.

SUPPLEMENTARY FILE 9 Reverse MR (psoriasis liability → gene expression).

SUPPLEMENTARY FILE 10 The correlation method used in Figure 6B and the corresponding *p*-values.

SUPPLEMENTARY FILE 11 The correlation method used in Figure 6D and the corresponding *p*-values.
