# Supplementary figures and images for: Potential marker genes for psoriasis revealed based on single-cell sequencing and Mendelian randomization analysis
Source: Front Genet. 2025 Nov 17;16:1634874. doi: 10.3389/fgene.2025.1634874 (PMC12665383; doi:10.3389/fgene.2025.1634874)

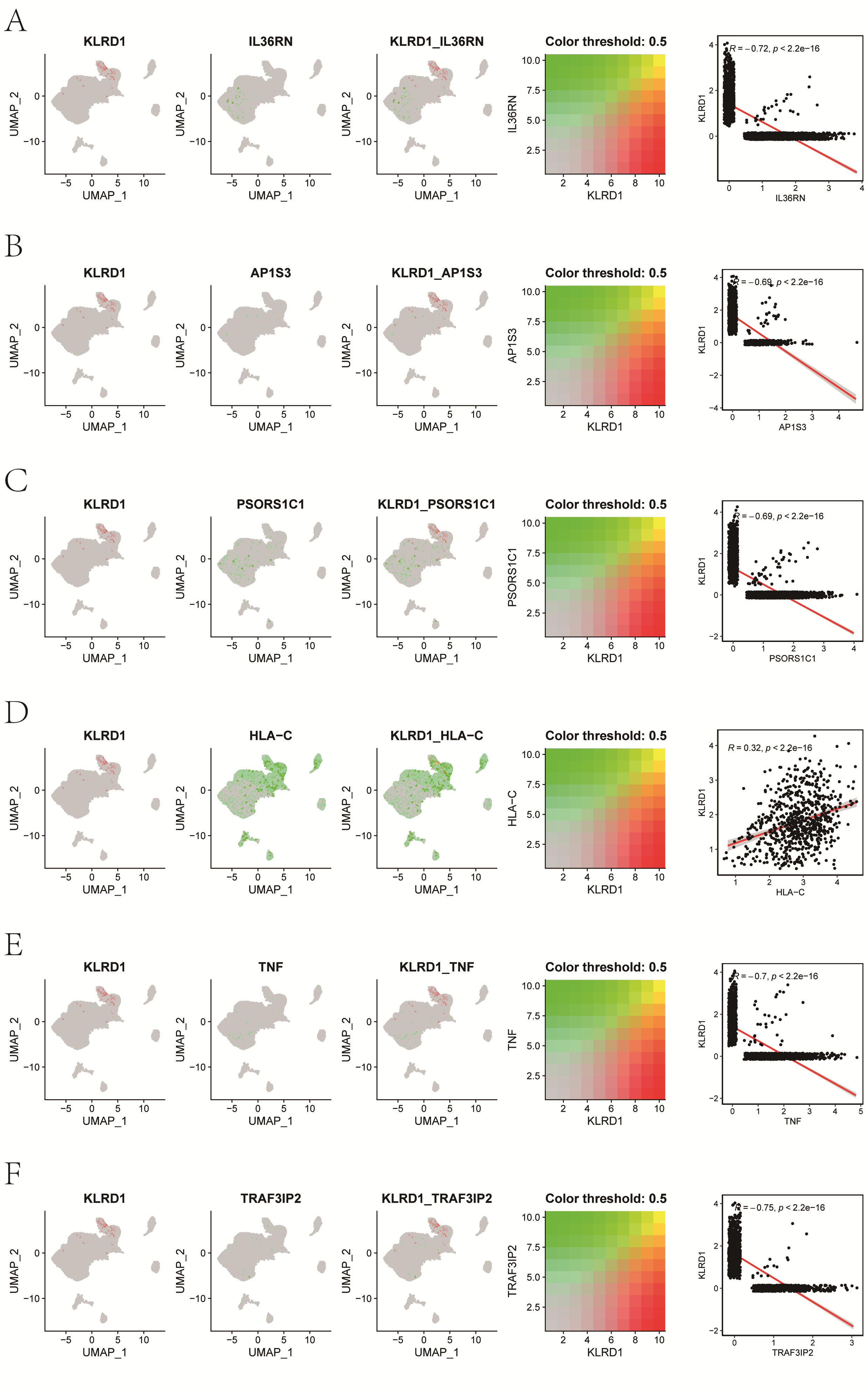

Supplement: Supplementary file 3 [file Image6.tif]

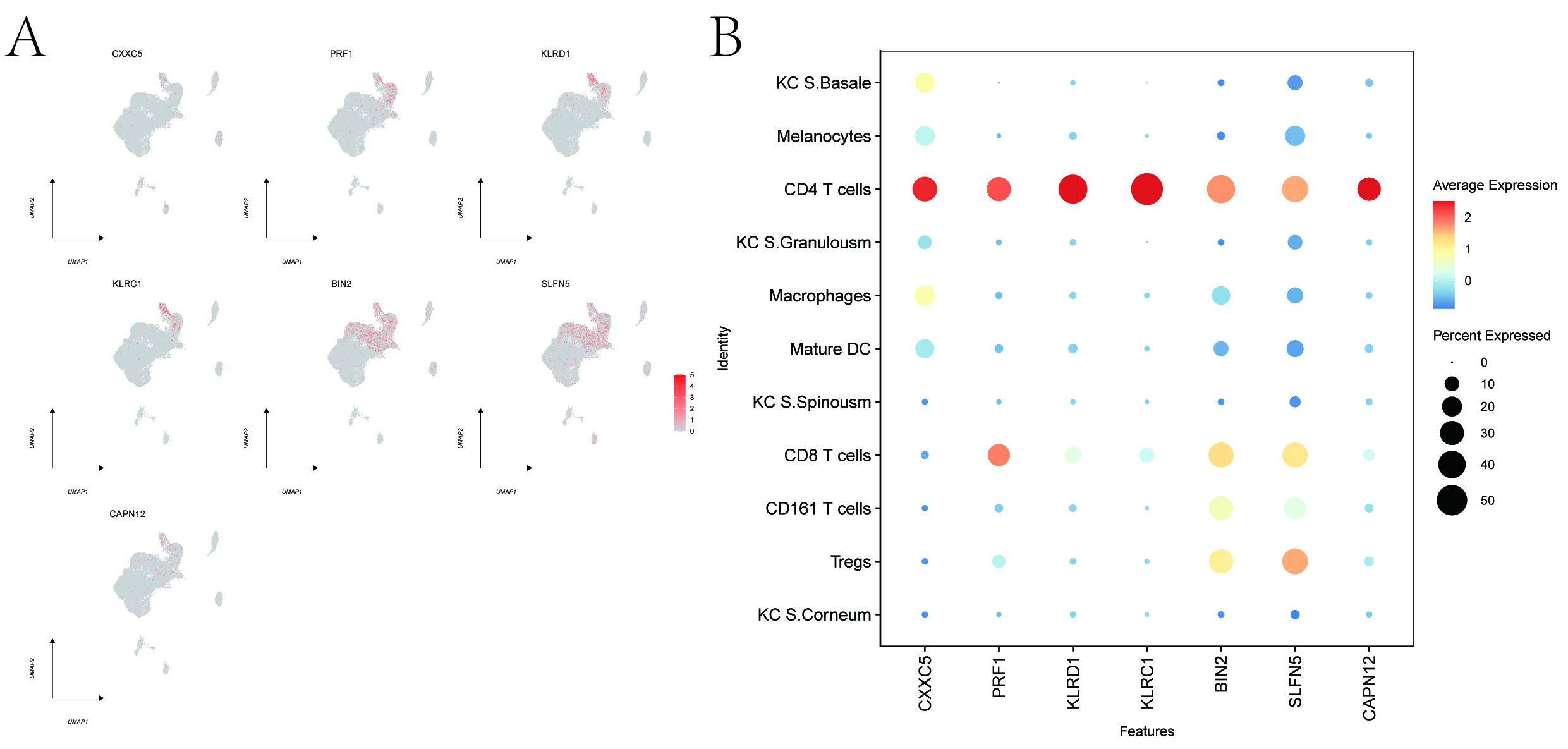

Supplement: Supplementary file 5 [file Image3.tif]

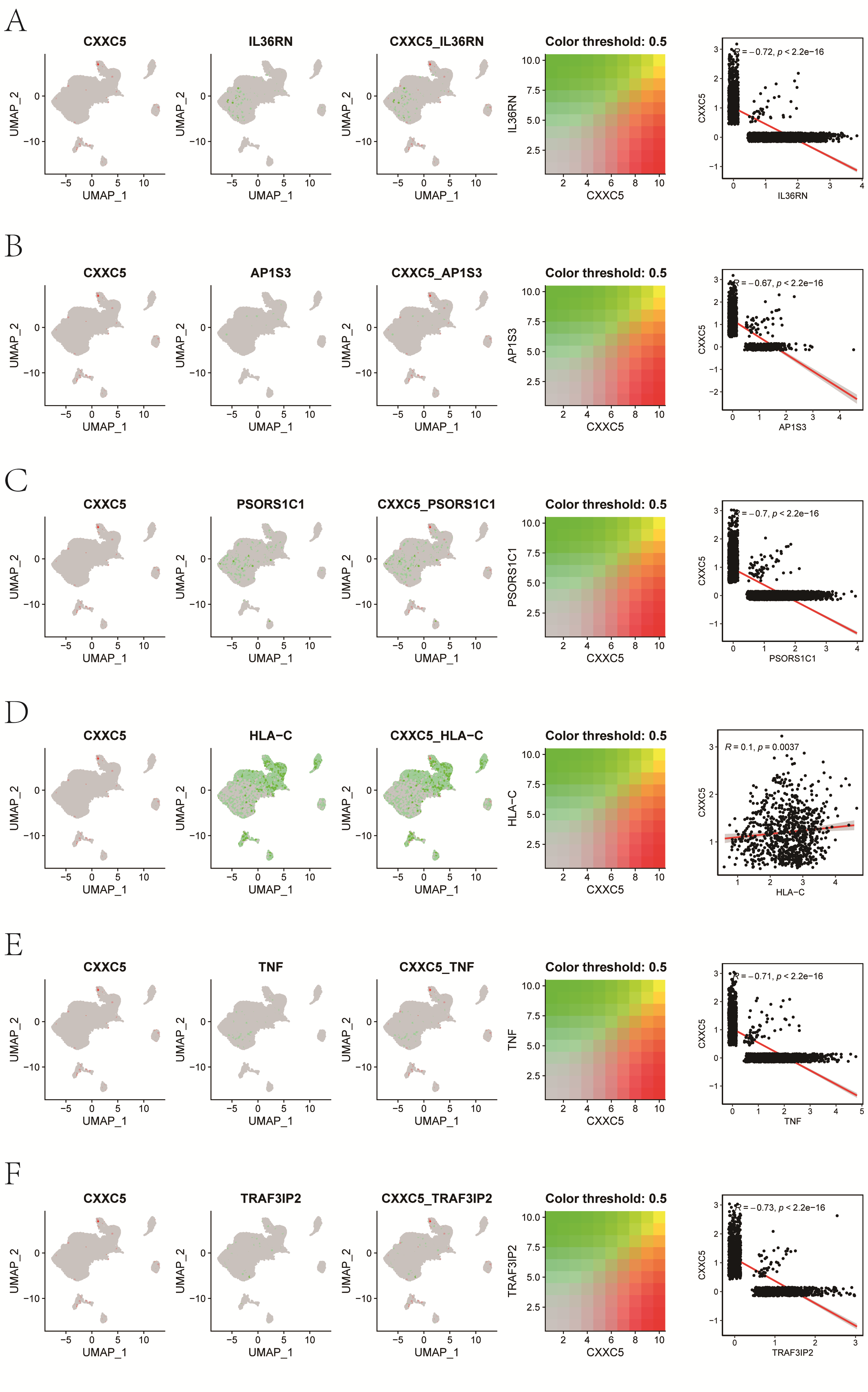

Supplement: Supplementary file 6 [file Image4.tif]

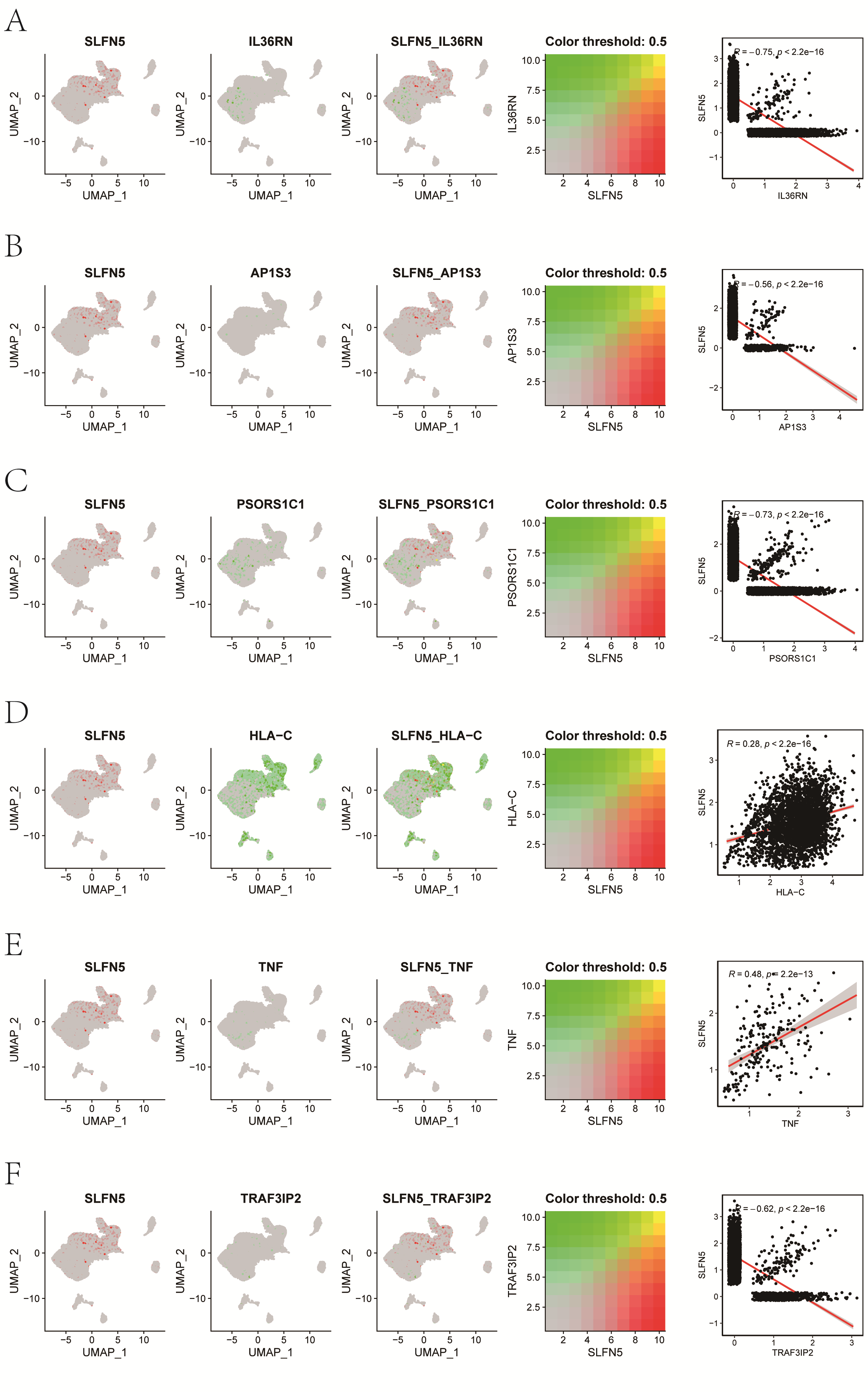

Supplement: Supplementary file 7 [file Image9.tif]

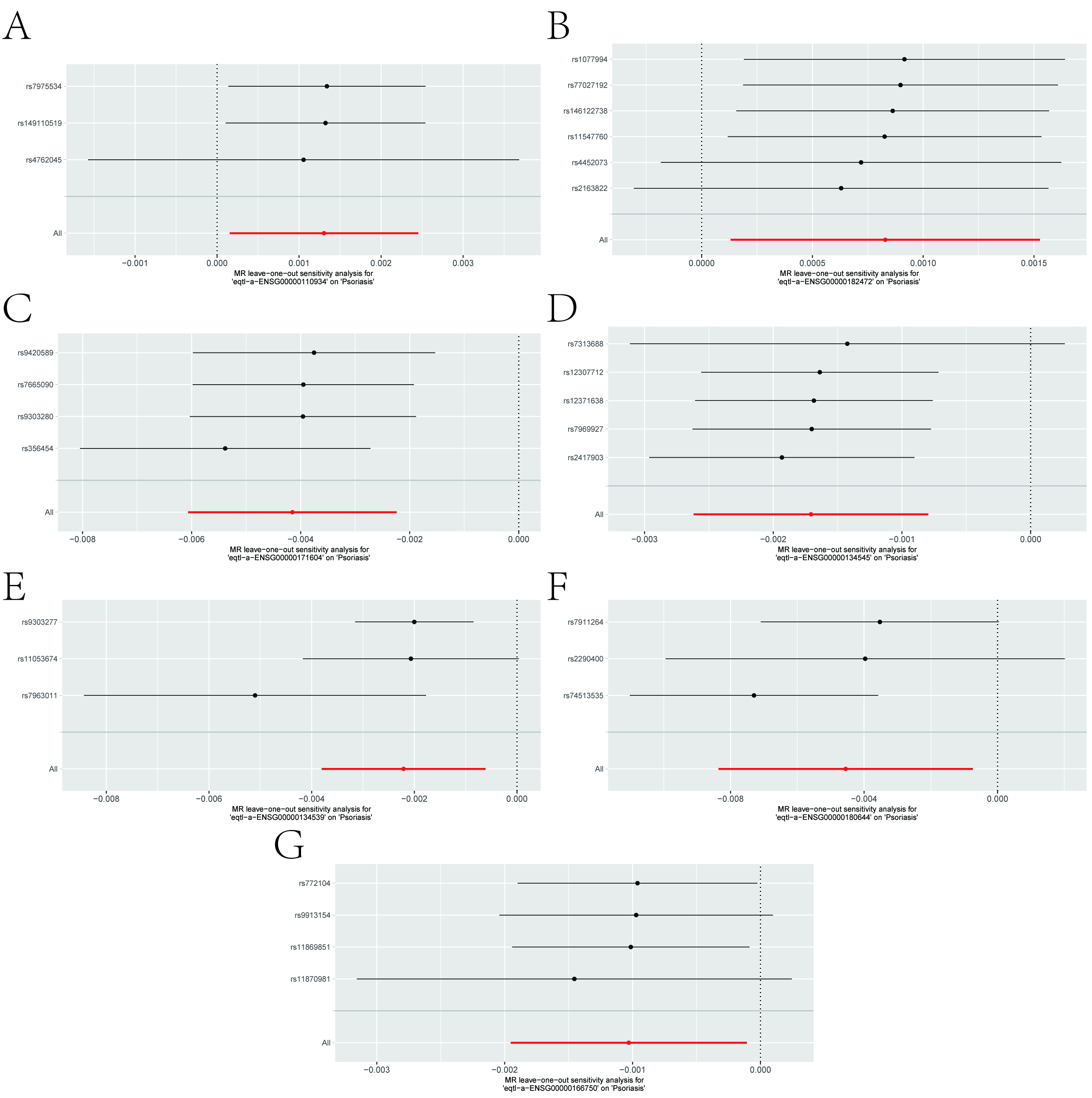

Supplement: Supplementary file 8 [file Image2.tif]

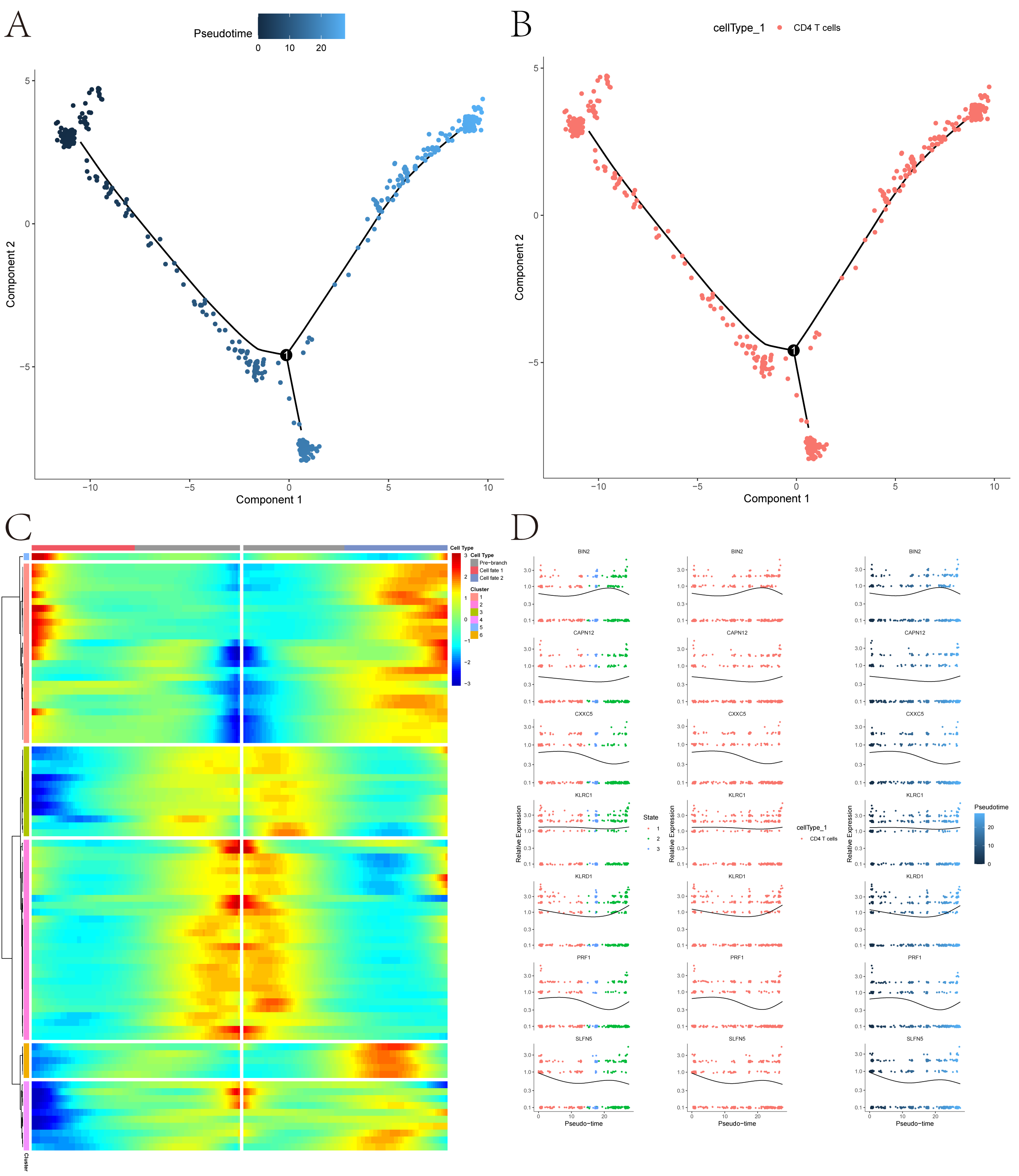

Supplement: Supplementary file 9 [file Image11.tif]

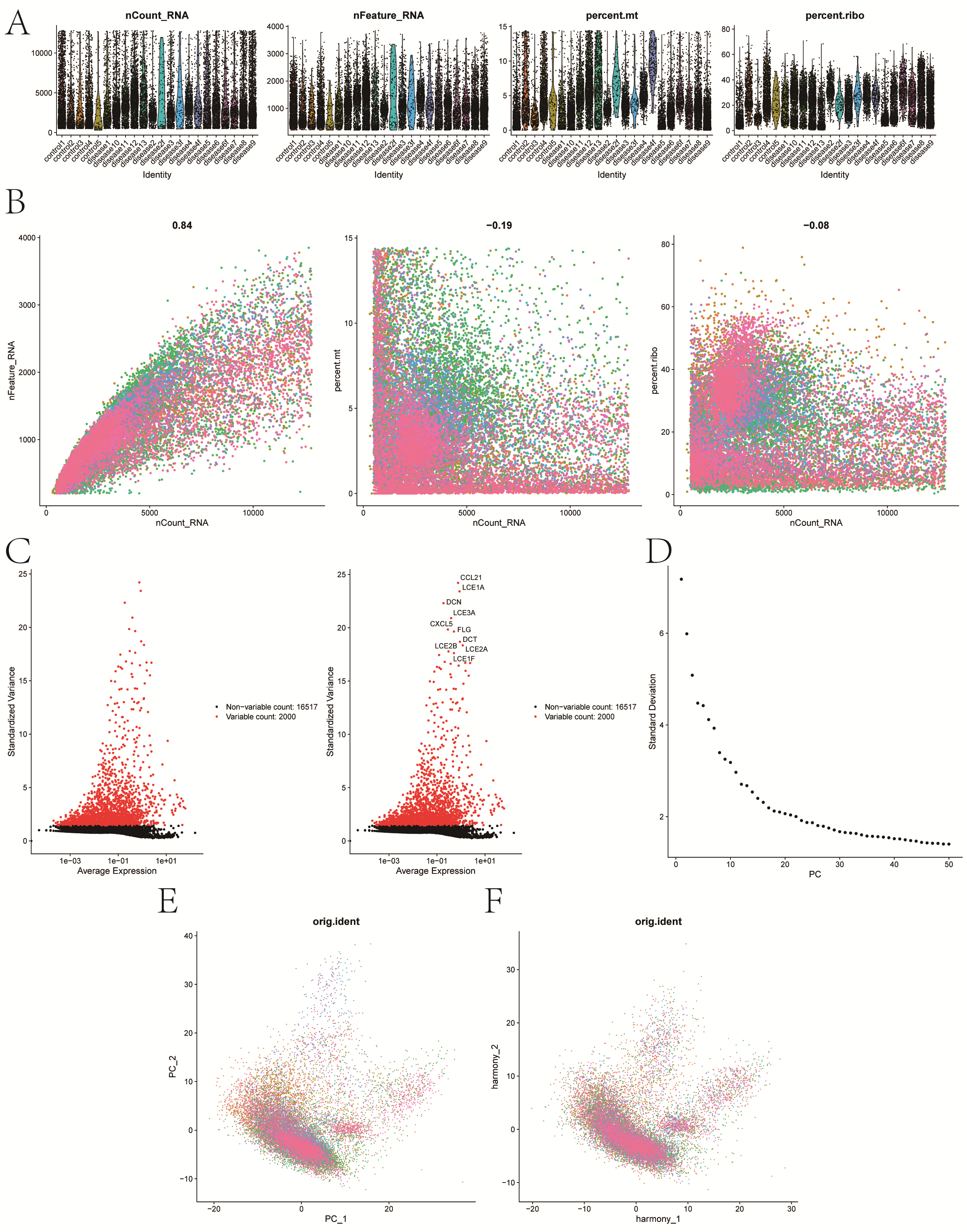

Supplement: Supplementary file 10 [file Image1.tif]

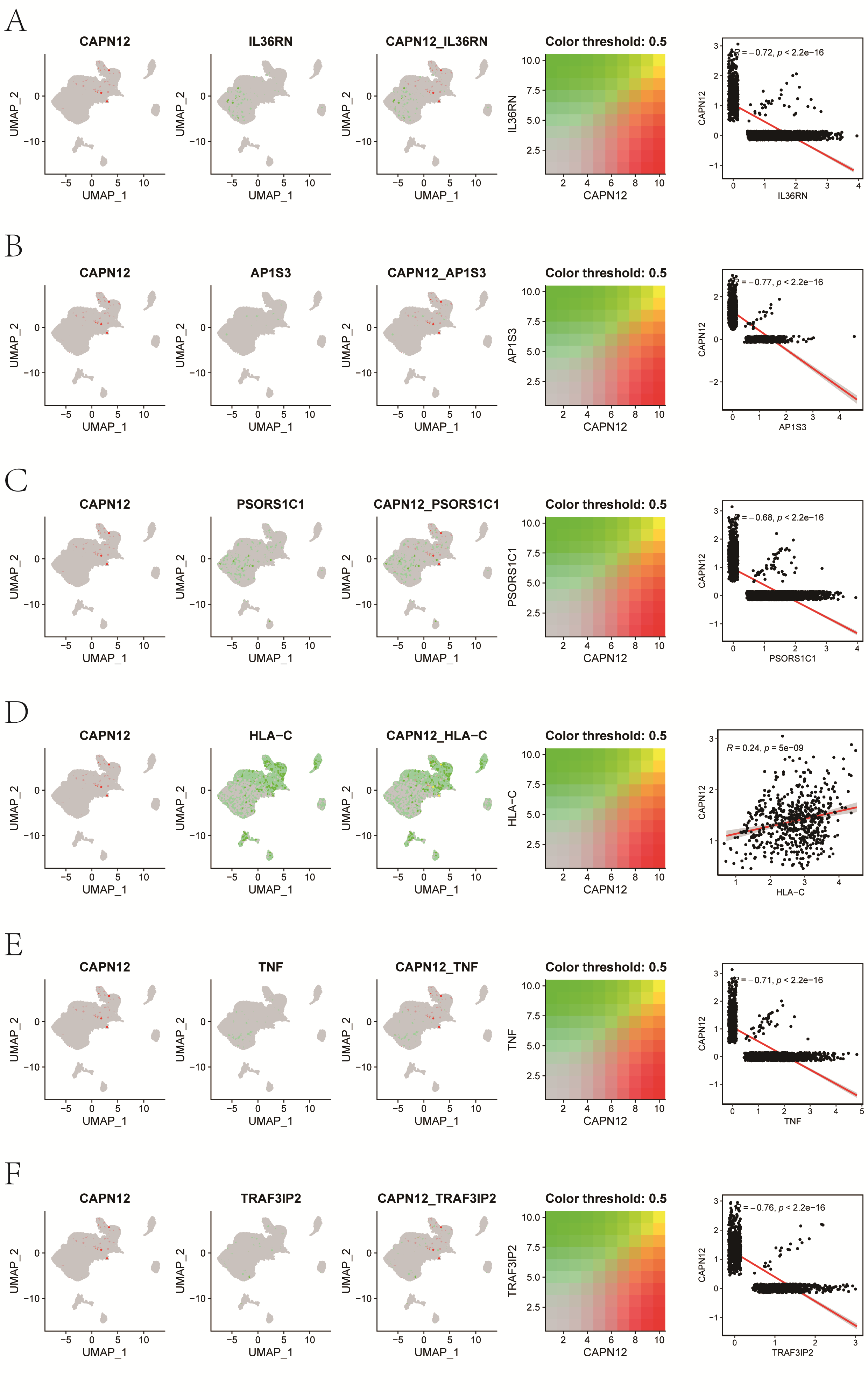

Supplement: Supplementary file 11 [file Image10.tif]

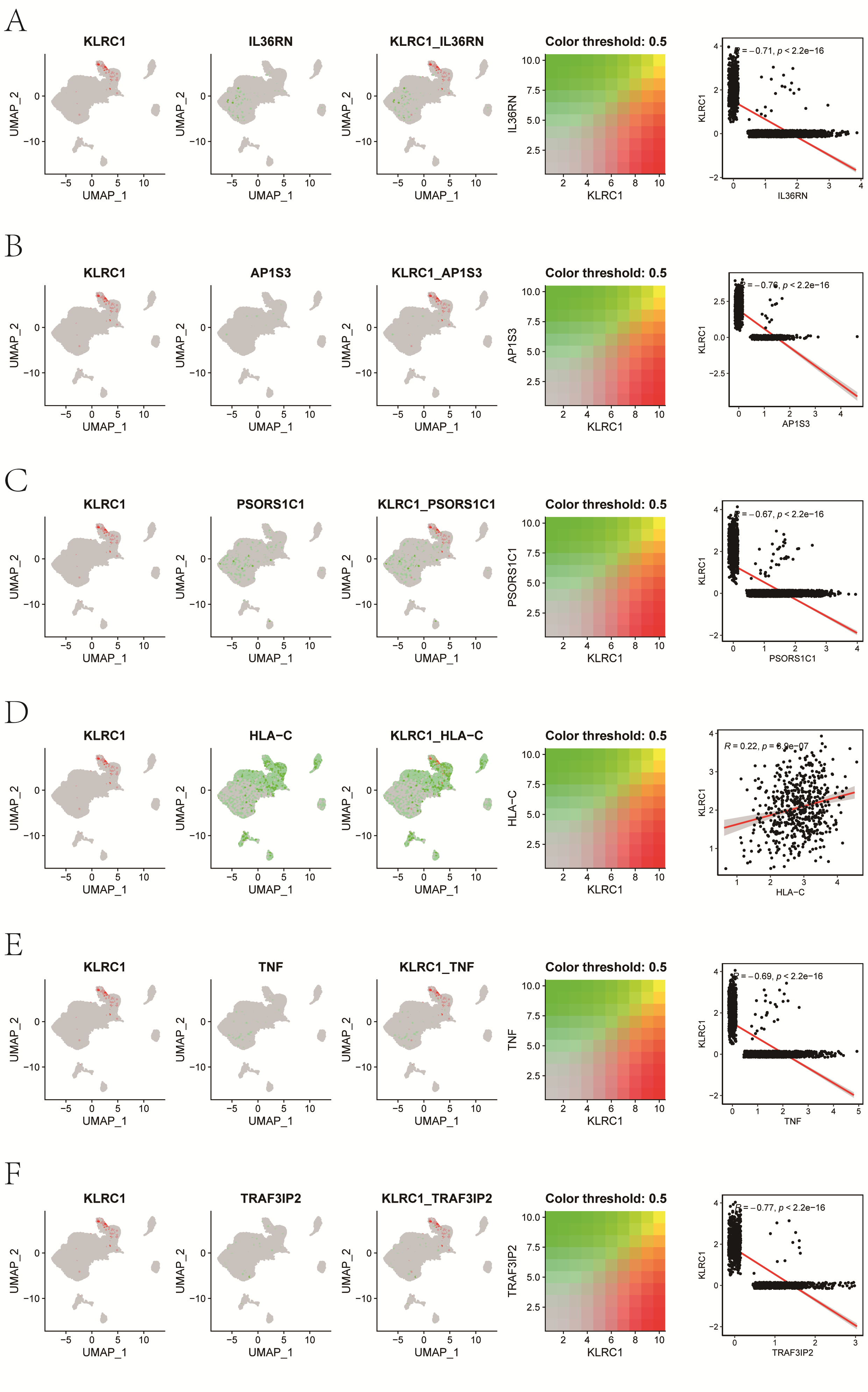

Supplement: Supplementary file 12 [file Image7.tif]

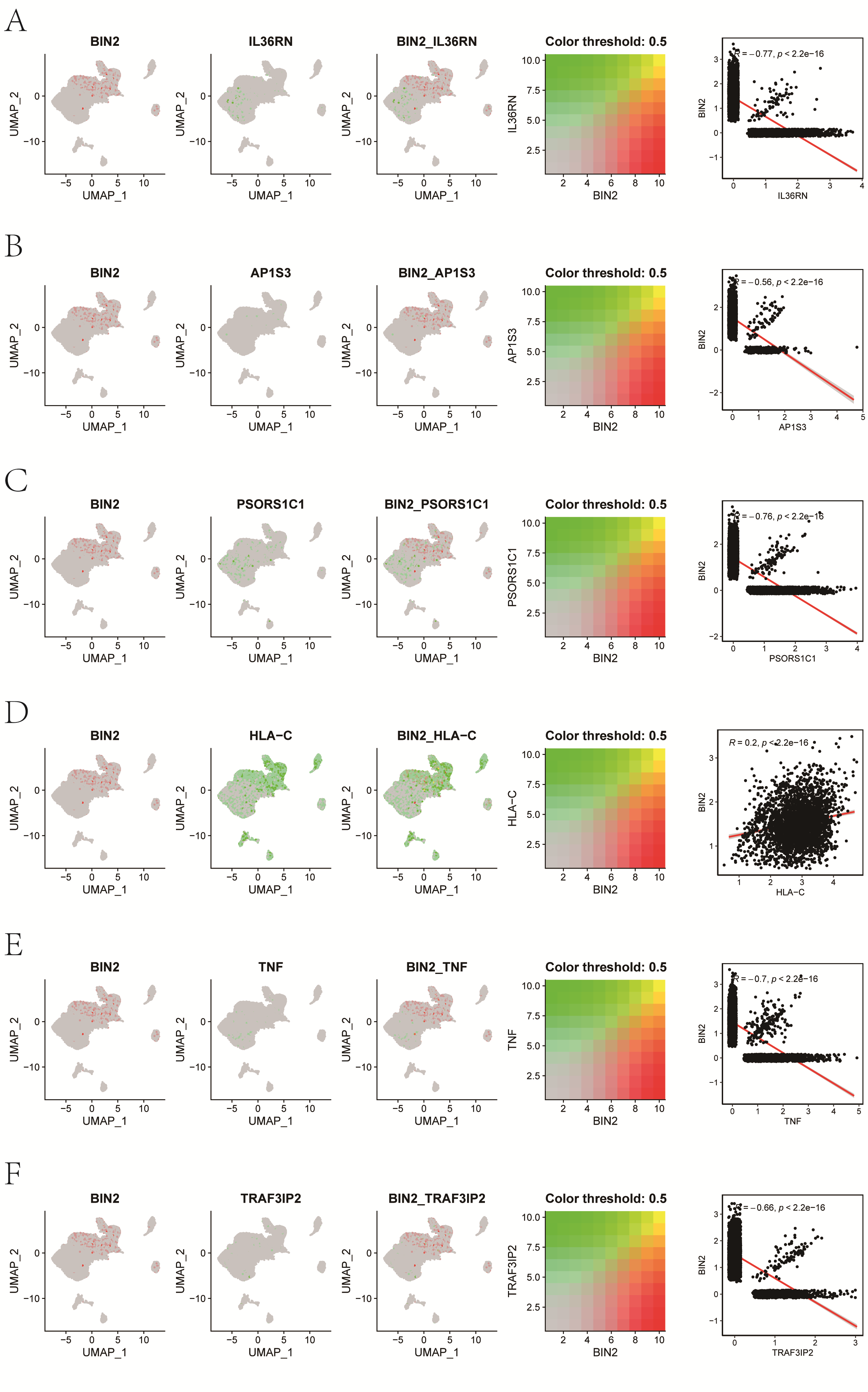

Supplement: Supplementary file 16 [file Image8.tif]

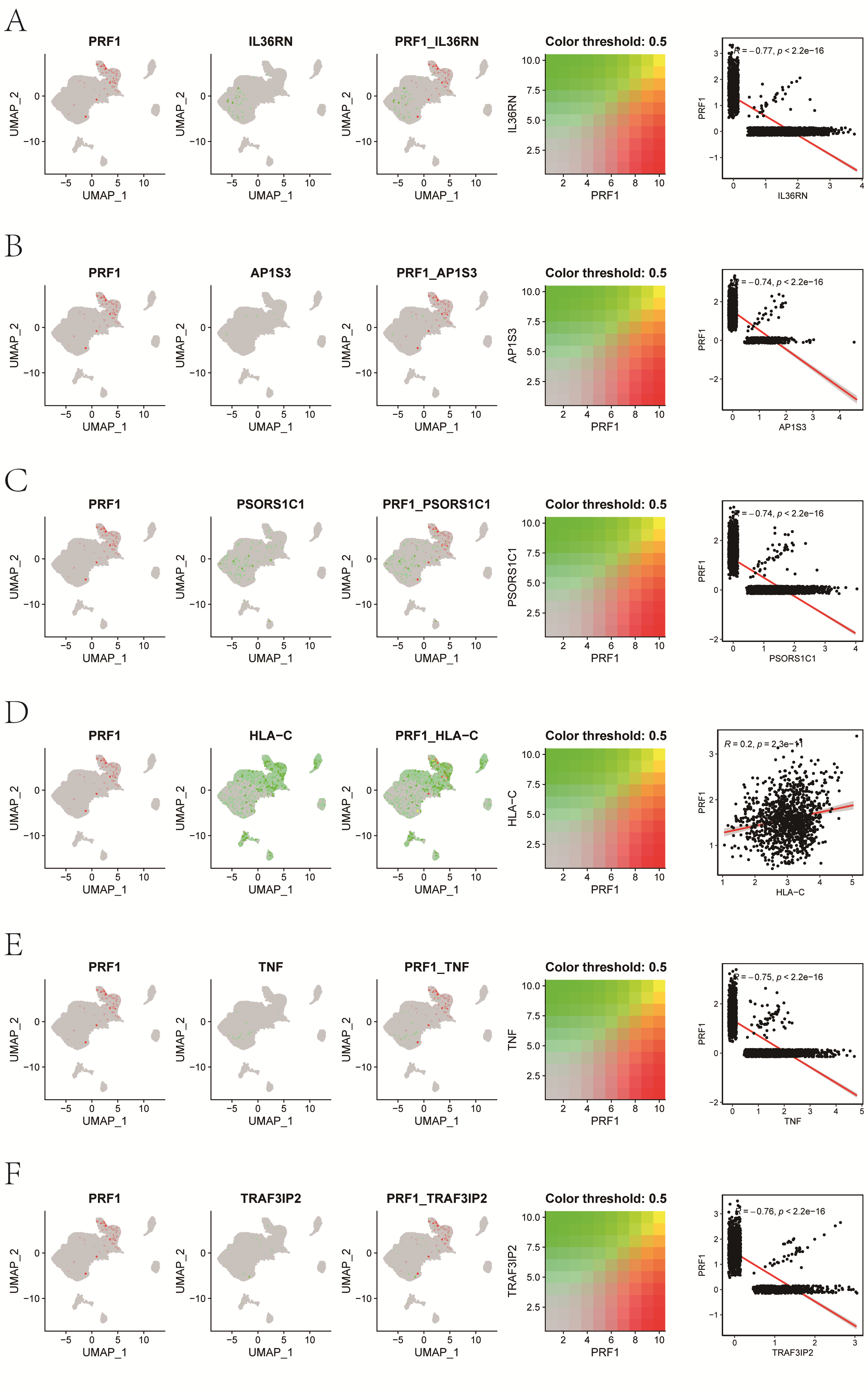

Supplement: Supplementary file 17 [file Image5.tif]
